# Implant for Augmentation of Cerebral Blood Flow Trial-1 (ImpACT-1). A single-arm feasibility study evaluating the safety and potential benefit of the Ischemic Stroke System for treatment of acute ischemic stroke

**DOI:** 10.1371/journal.pone.0217472

**Published:** 2019-07-03

**Authors:** Dheeraj Khurana, Subhash Kaul, Dietmar Schneider, Attila Csanyi, Ilona Adam, Nasli R. Ichaporia, Bernd Griewing, Laszlo Csiba, Attila Valikovics, Vinod Puri, Hans Christoph Diener, Stefan Schwab, Andreas Hetzel, Natan Bornstein, for the ImpACT-1 Study Group

**Affiliations:** 1 Postgraduate Institute of Medical Education and Research, Chandigarh, India; 2 Nizam’s Institute of Medical Sciences, Hyderabad, India; 3 Leipzig University, NICU & Stroke Unit, Leipzig, Germany; 4 Aladar Petz County Teaching Hospital, Gyor, Hungary; 5 Istvan Szechenyi University, Gyor, Hungary; 6 Jahangir Hospital, Pune, India; 7 Neurologische Klinik GmbH, Bad Neustadt, Germany; 8 University of Debrecen, Debrecen, Hungary; 9 B.A.Z.County Hospital, Miskolc, Hungary; 10 GB Pant Hospital, New Delhi, India; 11 Department of Neurology and Stroke Center, University Hospital Essen, Essen, Germany; 12 Universitat klinikum Erlangen, Erlangen, Germany; 13 University of Freiburg, Freiburg, Germany; 14 Tel Aviv Medical Center,Tel Aviv, Israel; University of Glasgow, UNITED KINGDOM

## Abstract

**Background:**

The Ischemic Stroke System is a novel device designed to deliver stimulation to the sphenopalatine ganglion(SPG).The SPG sends parasympathetic innervations to the anterior cerebral circulation. In rat stroke models, SPG stimulation results in increased cerebral blood flow, reduced infarct volume, protects the blood brain barrier, and improved neurological outcome. We present here the results of a prospective, multinational, single-arm, feasibility study designed to assess the safety, tolerability, and potential benefit of SPG stimulation inpatients with acute ischemic stroke(AIS).

**Methods:**

Patients with anterior AIS, baseline NIHSS 7–20 and ability to initiate treatment within 24h from stroke onset, were implanted and treated with the SPG stimulation. Patients were followed up for 90 days. Effect was assessed by comparing the patient outcome to a matched population from the NINDS rt-PA trial placebo patients.

**Results:**

Ninety-eight patients were enrolled (mean age 57years, mean baseline NIHSS 12 and mean treatment time from stroke onset 19h). The observed mortality rate(12.2%), serious adverse events (SAE)incidence(23.5%) and nature of SAE were within the expected range for the population. The modified intention to treat cohort consisted of 84 patients who were compared to matched patients from the NINDS placebo arm. Patients treated with SPG stimulation had an average mRS lower by 0.76 than the historical controls(CMH test p = 0.001).

**Conclusion:**

The implantation procedure and the SPG stimulation, initiated within 24hr from stroke onset, are feasible, safe, and tolerable. The results call for a follow-up randomized trial (funded by BrainsGate; clinicaltrials.gov number, NCT03733236).

## Introduction

In patients with acute ischemic stroke (AIS), reperfusion is associated with better neurological outcomes and anterograde reperfusion is the goal of current therapeutic strategies. Two direct reperfusion therapies have shown benefit in randomized trials and are recommended in guidelines for the management of eligible patients with AIS[1], but both have limitations. Intravenous (IV) thrombolysis use is limited by contraindications to lytic exposure in many patients, increased rates of hemorrhagic transformation compared to untreated patients, and low recanalization rates (30% of visualized artery occlusions)[2]. Endovascular thrombectomy (EVT) is constrained by being limited to large vessel occlusions accessible by thrombectomy devices, availability only at advanced thrombectomy-capable stroke centers, and risks of intracranial hemorrhage and infarcts in new territories[3–4].Therefore, there is a need for a therapy that is safe and efficacious in an extended time window, can be administered in frontline hospitals, does not require advanced imaging for patient selection, and is not associated with hemorrhagic transformation.

Enhancement of blood flow through collateral vessels is an attractive alternative or complementary therapeutic approach, yielding reperfusion of ischemic tissues via routes other than recanalization of the directly-supplying artery. AIS patients with good collateral flow have been shown to have lower rates of infarct expansion and improved functional outcomes[3–8].

The sphenopalatine ganglion (SPG) is the source of parasympathetic innervation to the anterior cerebral circulation[9]. In pre-clinical studies, SPG stimulation led to arterial vasodilatation, increased ipsilateral cerebral blood flow, and augmentation of cortical tissue perfusion[10]. In preclinical models of anterior circulation stroke, SPG stimulation reduced infarct volume [11,12], increased neuronal survival[13], reduced damage to the blood-brain barrier[10], and improved neurological outcome[10,13] when started at various time points, including up to 24 hours from stroke onset[10,13].

The goal of the ImpACT-1trial was to assess the feasibly, safety and tolerability of the implantation and of SPG stimulation. An additional goal was to evaluate the likelihood of potential benefit of SPG stimulation in anterior circulation AIS patients up to 24 hours from onset and inform the design of subsequent randomized trials.

## Methods

### Study design

Implant for augmentation of cerebral bloodflowtrial-1(ImpACT-1) was a prospective, multinational, single-arm, feasibility study. The study aim was to assess the safety and tolerability of SPG stimulation using the Ischemic Stroke System in patients with AIS in the anterior circulation, when this treatment was delivered within 24 hours of stroke onset. An additional goal was to evaluate the potential benefit of SPG stimulation and inform the design of subsequent randomized trials.

Ethical committees’ approvals were received starting June 2006 (a complete list of ethical committees and Ministries of Health approvals is provided in S1 File). Patients were enrolled between July 4^th^, 2006 to September 29^th^, 2008 in 14 centers in 4 countries. Last follow up visit was on January 2^nd^, 2009.

The study was not publicly registered before participant recruitment began as in 2006 registration was not mandated by the corresponding regulatory authorities. The study was retrospectively registered at clinicaltrials.gov (NCT03733236). The authors confirm that all ongoing and related trials for this intervention are registered.

The trial was designed by the sponsor, BrainsGate Ltd., which provided funding and the Ischemic Stroke System devices for the trial as well as central database maintenance. This does not alter the authors adherence to PLOS ONE policies on sharing data and materials.

Patients with baseline NIHSS 7–20 were screened for enrollment. Table 1 summarizes the main inclusion and exclusion criteria. Patients with massive stroke(defined as ≥2/3MCA territory involvement) were initially included in the study(in order to assess the safety profile of the treatment even with the most severe cases) and subsequently the protocol was amended and massive stroke was added to the exclusion criteria (due to the lack of comparable historical controls in the NINDS database).

**Table 1:** Inclusion and exclusion criteria.

Major inclusion and exclusion criteria
Inclusion
○ 18–85 years of age ○ Symptoms and signs of AIS within the anterior circulation ○ NIHSS ≥ 7 and ≤ 20 ○ Treatment could be initiated within 24 hours post stroke onset ○ Signed informed consent by the patient or legally authorized representative
Exclusion
○ Imaging diagnosis including tumor, abscess, primary intracranial hemorrhage (ICH) or secondary hemorrhage (PH1,PH2) (H1 and H2 were allowed); or symptoms suspicious for sub-arachnoid hemorrhage ○ Culprit lacunar infarct (unless brain imaging demonstrated a relevant lesion > 1.5 cm in size) ○ A stroke in the posterior circulation ○ Minor stroke or rapidly improving neurological symptoms with a high probability of a Transient Ischemic Attack (TIA) ○ Eligibility or treated with IV or IA rtPA or mechanical thrombectomy ○ NIHSS level of consciousness score ≥ 2 ○ Stroke in previous 6 months ○ modified Rankin Score > 2 before the stroke ○ Patients undertaking oral anticoagulants or having received heparin within 48 hours, and /or with elevated aPTT or INR ○ Septic embolus ○ Severe cardiac disease ○ Uncontrolled hypertension (systolic >185 mmHg and/or diastolic >110 mmHg) ○ Serious systemic infection ○ Pregnancy ○ Patients with other implanted neural stimulator ○ Orthodontics or non-Hygienic condition/ problems that prevent procedures within the mouth ○ Life expectancy < 1 year from other causes

Written informed consent by the patient or a legally authorized representative and IRB oversight were obtained. Once consent was obtained and eligibility was confirmed, the Implantable Neural Stimulator (INS) was implanted and SPG stimulation with the Ischemic Stroke System was initiated. The treatment consisted of 3–4 hours of SPG stimulation per day, for 5–7 consecutive days. At the end of treatment, the INS was removed, and the patients were followed for 90 days. Stimulation parameters were derived based on extensive preclinical studies (unpublished).Neurological assessments were performed at baseline and follow-up visits using the NIHSS.[14–17]Adverse events were monitored, evaluated and recorded over the study period. The study was approved by the ethics committee at all sites conducting the study and conducted in accordance with the provisions of the declaration of Helsinki and international standards for the conduct of clinical investigations of human subjects (S1 File).

### Device description

**Fig 1:**
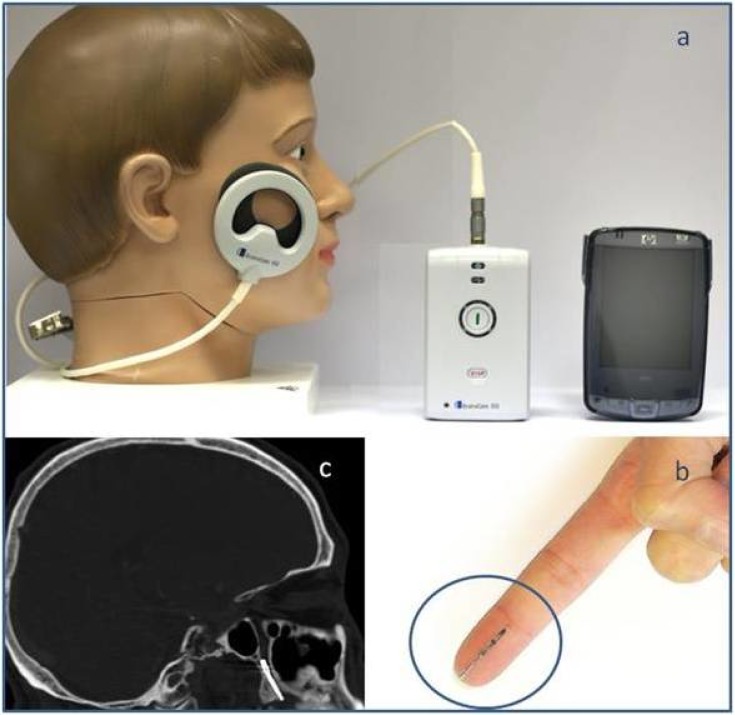
The ischemic stroke system. The energy delivery control subsystem (a) including the controller, driver and transmitter; the Implantable Neural Stimulator (b) and a sagittal view of a CT scan demonstrating the INS position (c).

The Ischemic Stroke System is a novel device intended for electrical stimulation of the SPG. It comprises two major components, the implantable neural stimulator(INS) (Fig 1B)and the energy delivery control subsystem(EDC) (Fig 1A).The INS is a 1-inch long implant inserted through the greater palatine canal using a minimally invasive oral procedure under local anesthesia. The INS is extra-cranially positioned with its distal tip located next to the SPG(Fig 1C). Activation of the INS electrically stimulates the SPG. In order to activate the implant, an external driver transmits RF-energy via a transmitter coil to an electronic circuit located in the proximal end of the implant(Fig 1A).The transmitter is attached to the patient’s cheek with a disposable, single use sticker for the duration of the treatment and removed after each treatment session. The transmitter receives constant feedback streams from the implant, indicating correct operation and signaling in case of signal failure and the need for repositioning the transmitter. A handheld computer (Fig 1A) serves as controller allowing the physician to control and monitor the treatment status. A detailed description of the Ischemic Stroke System components has previously been described[18]. The INS is removed simply by locating and gently pulling a short excess thread which is left during implantation adjacent to the canal opening.

### End points and statistical method

Patients were evaluated at baseline, day7 (after last treatment session), 30±5and 90±5 days poststroke onset. Missing data was completed using last observation carried forward (LOCF). The primary endpoint was assessing the safety of the device implantation and treatment. Safety outcomes were: 1) mortality; 2)serious adverse events; 3) procedural complications and device-related adverse events; and 4)the need to stop treatment sessions.

Signal of potential benefit was measured at 90 days to facilitate the design of future randomized trials. As the study was a single arm study, comparison was performed post-hoc with a historical control population. This group was composed of the placebo group of the NINDS rtPA studies I and II [19].From this group,165 patients matched the study’s NIHSS range of 7–20 at 24 hours from stroke onset (Table 2). Mean age was 66.3(±11.7) years, 41% females (67/165), median NIHSS was 13 IQR (10–16).The NIHSS score at 24hours was used in order to match the time from stroke on set between populations. Secondary outcomes were: 1) the shift in the distribution of modified Rankin Scale (mRS) scores; 2) a dichotomized mRS (defined as the proportion of positive outcomes, where a positive outcome was an mRS score between 0 and2); 3) binary NIHSS, defined as the proportion of positive outcomes assessed using the NIHSS scale, where positive outcome was a complete recovery (NIHSS 0 or1) or an improvement by 9 or more points in the NIHSS score, when comparing day 90 to the baseline and 4) Barthel index.

**Table 2:** Baseline characteristics by sub-population.

	All	Not Treated	Treated, massive stroke	Treated, non-massive stroke	Historical Controls
	N = 98	N = 6	N = 7	N = 85	N = 165
Mean age (±SD), years	56.75 (±14.97)	48 (±8.40)	58.43 (± 12.2)	57.2 (±15.45)	66.3 (±11.7)
Left hemisphere stroke %	52%	50%	43%	53%	44%
Female %	34.7%	16.7%	28.6%	36.5%	40.6%
Asian Indians %	76.5%	83%	100%	74%	<2%
European Caucasians %	23.5%	17%	0%	26%	63%^(*)^
Time since stroke onset, mean (±SD), hours	18.6 (±5.1)	21.15 (±3.32)	12.61 (±5.11)	19.02 (±4.74)	24^(**)^
Time since stroke onset, median (min-max), hours	18.5 (5–27)	21.15 (18.8–23.5)	12.75 (5–23.50)	19.0 (8–27)	24^(**)^
Baseline NIHSS, mean (±SD)	12.23 (±3.27)	12.00 (±3.22)	15.00 (±2.08)	12.02 (±3.27)	13.2 (±3.8)
Baseline NIHSS, median	12.0	11.5	16.0	12.0	13

(*) Categorized as “White, non-Hispanic” in NINDS

(**) Time from onset to baseline NIHSS measurement

The modified Intent to Treat (mITT) cohort was defined as patients who received at least one treatment session and had at least one mRS follow up. Statistical analysis was done using JMP 5 (SAS institute). Outcome measures aimed to assess potential benefit were tested for statistical significance using the van-Elteren version of the Cochran-Mantel-Haenzel test (CMH), and the Chi Square test. Significance was defined using an alpha(type-I error) level of 0.05.

## Results

**Fig 2:**
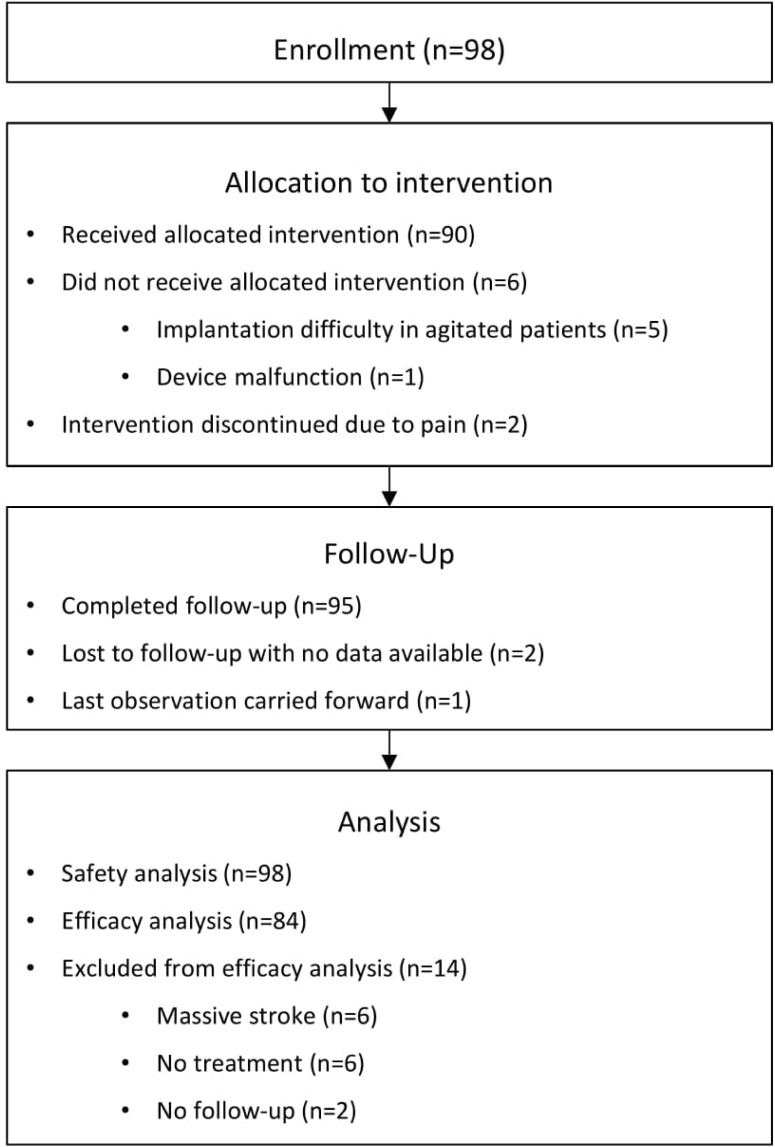
CONSORT flowchart.

A total of 98patients were enrolled in ImpACT-1(Table 2).The mean age was 56.8 year; 52.0%(51/98) had left hemispheric strokes; 34.7%(34/98) were female;76.5%(75/98) were Asian Indians and 23.5% (23/98) were European Caucasians; the mean baseline NIHSS score was 12.2; the mean time from stroke onset(TFSO) was 18.6 hours. Six patients (6.1%) were not treated (Fig 2),five of whom(5.1%) due to device implantation difficulties in agitated patients. In one case (1.0%), the treatment was not initiated due to device malfunction. This small population was comprised of relatively young patients with no significant differences in stroke severity (mean baseline NIHSS 12.0).

Two sub-populations comprised the group of treated patients. Seven patients (7.1%) had massive strokes (defined as ≥2/3MCA territory involvement). The other 85 patients (86.7%) had non-massive stroke (<2/3 of MCA territory, Table 2). Baseline characteristics of the massive subgroup indicated a higher stroke severity compared to the non-massive subgroup(mean baseline NIHSS of 15 and 12,respectively) and a shorter TFSO (12.75 hours and 19 hours, respectively).Two treated patients (2.0%)were lost to follow up.

### Safety

Twelve patients (12/98;12.2%) died during the study. Eight patients were from the non-massive (8/85;9.4%); three patients were from the massive subgroup (3/7;42.9%) and one patient(1/6, 16.7%) was from the non-treated subgroup(Table 3).The patients who died were older than the overall population and had more severe strokes as measured by baseline NIHSS. In 7 of the 12 patients (58%), the primary cause of death was cardiovascular. In 3 of the 12 patients (25%),the primary cause of death was related to the stroke (including 2 of 3 from the massive subgroup).Two of the 12 patients (16.7%) died of respiratory infections. All deaths were defined by the investigators as not related to the treatment. The incidence of mortality in the study(12.2%) compares favorably to that reported for the matched historical controls from the NINDS trial (19.4%).

**Table 3:** Serious adverse events (fatal).

	MS (massive stroke) pts	NMS (non-massive stroke) pts	Not Treated	Total^(*)^
	N = 7	N = 85	N = 6	N = 98
Cardiovascular		6(7.1%)	1 (16.7%)	7 (7.1%)
Stroke Related	2 (28.6%)	1 (1.2%)		3 (3.1%)
Respiratory Infections	1 (14.3%)	1 (1.2%)		2 (2.0%)
Total	3 (42.9%)	8 (9.4%)	1 (16.7%)	12(12.2%)

^(*)^ % represents percentage of patients

At least one SAE(including death) was reported in 23 patients (23.5%).The three most frequent SAE etiologies were cardiovascular, nervous system and respiratory events (Table 4).Three SAE were classified as related or possibly related to the device, including one case of sedation resulting in intubation in an uncooperative patient during INS removal; one case of hemorrhagic transformation in a patient who suffered from atrial fibrillation and received both Clopidogrel and Aspirin following hospitalization; and one case of neurological deterioration. Other SAEs were not considered by the investigators to have a relationship to treatment.

**Table 4:** Serious adverse events (non-fatal).

	MS (massive stroke) pts	NMS (non-massive stroke) pts	Total
	N = 7	N = 85	N = 98
Cardiovascular	3	8	11(11.2%)
Nervous system	5	7	12(12.2%)
Respiratory system	7	1	8 (8.2%)
Other	7	5	12(12.2%)
Total	22	21	43(23.5%)

Non-serious device-related adverse events are detailed in Table 5. Treatment related AEs included 3 patients (3.1%) who under went re-implantation and 5 patients (5.1%) who were diagnosed with implant misplacement on CT. Four patients (4.1%) reported pain in the implantation site. No patient suffered a permanent sensory deficit. No serious bleeding or infection were reported.

**Table 5:** Procedure complications and device-related adverse events (Non-serious).

	Number of patients reported	% of patients reported (N = 98)	Number of Events Reported
Pain during stimulation	15	15.3%	25
Lacrimation ^(*)^	8	8.2%	8
Complication of device insertion	5	5.1%	5
Implant misplacement	5	5.1%	5
Implantation site pain	4	4.1%	4
Re-implantation	3	3.1%	3
Implantation site inflammation	3	3.1%	3
Redness of the face ^(*)^	3	3.1%	3
Device accidentally removed by patient	2	2.0%	2
Salivation ^(*)^	2	2.0%	2
Device malfunction	1	1.0%	1
Paresthesia	1	1.0%	1
Implantation site bleeding upon removal	1	1.0%	1
Wound dehiscence	1	1.0%	1

^(*)^ Expected surrogates of SPG activation, typically not be reported as adverse reactions.

### Tolerability

Overall, the treatment was well tolerated. The most frequent adverse event reported was sensation or pain during stimulation, which was reported in 15/92 (16.3%) subjects as a mild event that resolved in the majority of cases without treatment and/or stimulation discontinuation. Sensation or pain during stimulation was an anticipated physiological response due to the involvement of sensory fibers known to pass through the SPG and the inability to adjust the stimulation level using the first generation of the device. Other visible physiological responses to the stimulation included lacrimation, salivation and redness of the face during stimulation, were all mild in nature and clinically insignificant.

In two cases (2.2%), stimulation was discontinued due to pain. The device design was then modified to enable the adjustment of the stimulation level, reaching a tolerable level for each individual.

### Signal of potential benefit

This hypothesis-generating analysis was performed on the mITT cohort, which included 84 patients (85.7%)–see CONSORT chart in Fig 2. The remaining 14 patients (14.3%) were excluded from the mITT cohort for the following reasons:6 massive stroke patients (6.1%) initially included to support safety analysis (one massive stroke patient was included in them. ITT cohort since he was recruited after the protocol was amended to exclude massive strokes); 6 patients (6.1%) were not treated;2 patients (2.0%) were lost to follow up with no available data. For a single patient (1.0%), last observation carried forward was performed using day 7 assessment.

**Fig 3:**
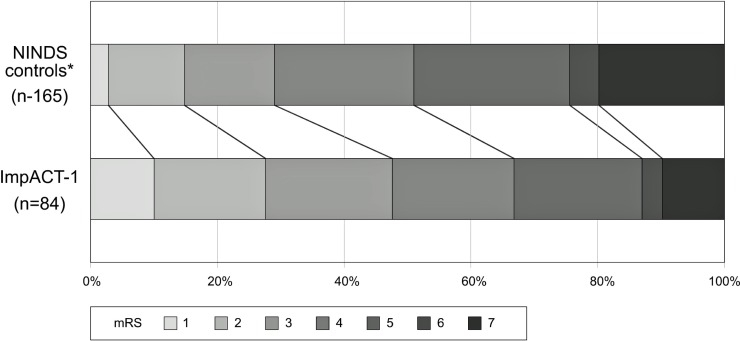
mRS shift. Outcome on day 90 expressed as the distribution of mRS scores in ImpACT-1 vs. NINDS control group (p = 0.001). * All NINDS control patients recruited with a 7–20 NIHSS at 24h.

**Fig 4:**
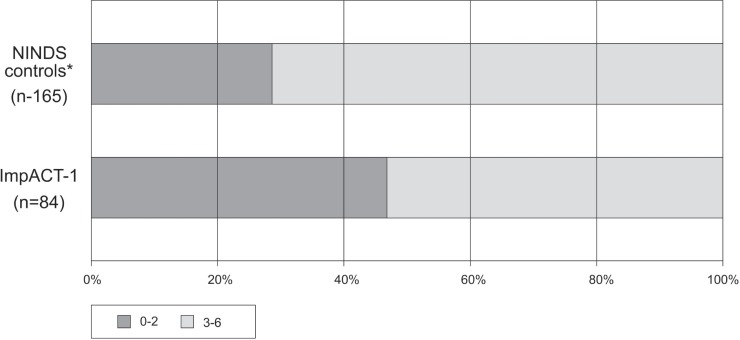
Dichotomized mRS. Outcome on day 90 expressed by dichotomized mRS scores in ImpACT-1 vs. NINDS control group (p = 0.004).* All NINDS control patients recruited with a 7–20 NIHSS at 24h.

**Fig 5:**
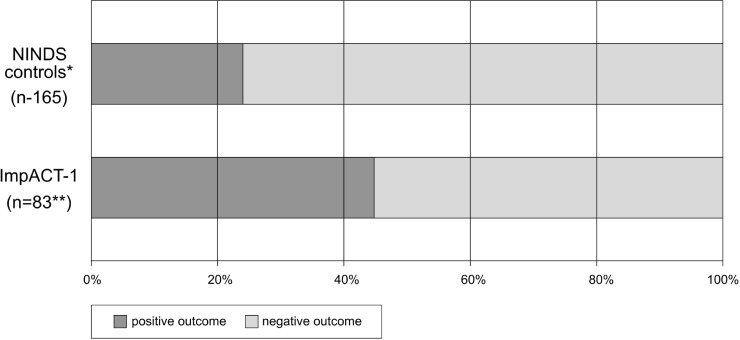
Binary NIHSS. Outcome on day 90 expressed by binary NIHSS scores in ImpACT-1 vs. NINDS control group (p = 0.0006).* All NINDS control patients recruited with a 7–20 NIHSS at 24h. Day 90 NIHSS score is not available for one of the 84 mITT patients.

In Figs 3–5, outcomes of ImpACT-1 patients were compared to the NINDS historical controls. ImpACT-1 patients were younger than the NINDS controls (mean age 56.75±14.97 vs 66.3±11.7 years), had higher proportions of left hemisphere strokes (52% vs. 44%) and different ethnic population distribution (Asian Indians: 76.5% vs. <2%). The average mRS difference was 0.76 points lower in ImpACT-1 compared to the NINDS controls (CMH test p = 0.001).The rate of functional independence (mRS 0–2) was 48% (40/84) in ImpACT-1 compared to29% (48/165) in the NINDS controls(p = 0.004). The binary NIHSS success rate was 45%(38/84) in ImpACT-1 compared to 23.6%(39/165) in the NINDS controls (p = 0.0006). Rates of completion of the Barthel index were low (<75%), so this endpoint was not formally analyzed.

An additional post-hoc analysis was performed on patients with aphasia. Thirty patients from the mITT cohort (39/84, 35.7%) entered the study with aphasia, as defined by a baseline score of more than 0 on item 9 of the NIHSS scale. Patients with aphasia had higher baseline NIHSS compared to patients without aphasia (average13.5versus 10.9) and a greater proportion of left-side stroke (24/30, 80% vs 20/54, 37%). A lower mortality rate in patients with aphasia was observed compared to patients without aphasia (2/30, 6.7% vs. 6/54, 11.1%) and better outcome as measured by binary NIHSS (19/30, 63.3% vs. 19/54, 35.1%;p = 0.05). A similar trend was observed in the 90-day dichotomized mRS (16/30, 53.3% vs. 24/54, 44.4%).

## Discussion

This feasibility study evaluated for the first time the use of a novel implantable device (the Ischemic Stroke System) for the treatment of AIS in the anterior circulation upto 24 hours after stroke onset. Results demonstrate safe and tolerable implantation procedure and treatment, and a safety profile comparable with previous studies (12.2%mortality;23.5%SAEs). Despite the known limitations of comparisons with historical controls, this comparison demonstrated a possible signal of potential benefit that warrants further investigation in randomized trials.

This study has several limitations. First, the study is a single-arm study and lacks a direct control group with sham stimulation. The use of the NINDS placebos as a historical control may limit the generalization of the results to current day controls, due to differences in standard of care and potential differences in ethnicity and etiology, and due to imbalance in baseline characteristics between the study population and the historical controls (younger age, higher proportion of left-hemisphere strokes, differences in ethnicity, and earlier measurement of baseline NIHSS in the study). Second, the relationship of SAEs to the treatment, as well as the clinical outcome measures, were adjudicated by unblinded investigators, this being a single-arm trial. Third, the potential clinical outcome analyses (comparison to the NINDS historical controls, the massive strokes analysis and the aphasic patient’s analysis) were performed post-hoc, and Fourth, the publication of this full report of study results was unduly delayed from the time of the final follow-up visit.

Implantation as well as stimulation sessions presented no significant safety concerns. Sensations during stimulation were an anticipated physiological response due to the involvement of sensory fibers known to pass through the SPG. These observations prompted the development of a sensation-based blinding mechanism which will be implemented in the following pivotal study. Mortality as well as SAE rates were comparative to those reported in other recent clinical trials[3,4]. A small subgroup of patients with massive stroke was included. Mortality in these patients (42.9%; 3/7) compares favorably to the expected mortality in this population[20]. A low incidence of neurological SAEs was demonstrated.

The post-hoc subgroup analysis of patients with aphasia is of particular interest. While patients with aphasia have been reported to suffer from higher mortality rates and worse neurological prognosis[21], the current study demonstrates an enhanced effect of SPG stimulation in patients with aphasia. This may be because aphasia is a cortical symptom, where most of the leptomeningeal collateral vessels are concentrated. The relatively long TFSO provides hope that SPG stimulation may offer a much-needed broader therapeutic time window for stroke treatment and allow therapy delivery to a significantly larger population.

## Conclusion

ImpACT-1 evaluated a novel device for the treatment of AIS in the anterior circulation upto 24 hours from stroke onset. In this single arm study, we confirmed feasibility of the intervention and did not identify significant safety concern that precludes further study. In comparison to historical controls functional outcomes were better in people treated with the Ischemic Stroke System. This will be formally assessed in the multinational, randomized, double blind, sham controlled pivotal study (IMPACT 24). Source of Funding: The trial was supported by BrainsGate,Ltd.

## Supporting information

S1 Dataset Study patients dataset. Records of all patients recruited to the study.
(DOC)

S1 File Ethics committees. List of all ethics committees, IRBs and Ministries of Health approval dates.
(PDF)

S2 File ImpACT-1 TREND checklist. Trend statement checklist.
(PDF)

S3 File STROKE protocol CLP1000450. Complete study protocol and clinical investigation plan.
(PDF)
