# Supplementary material for: Implant for Augmentation of Cerebral Blood Flow Trial-1 (ImpACT-1). A single-arm feasibility study evaluating the safety and potential benefit of the Ischemic Stroke System for treatment of acute ischemic stroke
Source: PLoS One. 2019 Jul 3;14(7):e0217472. doi: 10.1371/journal.pone.0217472 (PMC6609146; doi:10.1371/journal.pone.0217472)
Supplement: S1 Dataset — Records of all patients recruited to the study. (DOC) [file pone.0217472.s001.doc]

**Supplemental Data**

|  | Massive * | Non  Massive | Not  Treated | Total |
| --- | --- | --- | --- | --- |
| Cardiovascular |  | 6 | 1 | 7 (7.1%) |
| Stroke Related | 2 | 1 |  | 3 (3.1%) |
| Respiratory  Infections | 1 | 1 |  | 2 (2.0%) |
| Total | 3 (42.9%) | 8 (9.4%) | 1 (16.7%) | 12 (12.2%) |

*Massive stroke was defined by imaging (CT or Diffusion MR), as a lesion that occupies more than two thirds of MCA territory.

**Table 3**: Serious Adverse Events ( Deaths)

|  | Massive | Non  Massive | Total |
| --- | --- | --- | --- |
| Cardiovascular | 3 | 8 | 11 (11.2%) |
| Nervous system | 5 | 7 | 12 (12.2%) |
| Respiratory system | 7 | 1 | 8 (8.2%) |
| Other | 7 | 5 | 12 (12.2%) |
| Total | 22 | 21 | 43 (12.2%) |

**Table 4: Serious Adverse Events (Non-Death)**
