# Supplementary material for: Implant for Augmentation of Cerebral Blood Flow Trial-1 (ImpACT-1). A single-arm feasibility study evaluating the safety and potential benefit of the Ischemic Stroke System for treatment of acute ischemic stroke
Source: PLoS One. 2019 Jul 3;14(7):e0217472. doi: 10.1371/journal.pone.0217472 (PMC6609146; doi:10.1371/journal.pone.0217472)
Supplement: S1 File — List of all ethics committees, IRBs and Ministries of Health approval dates. (PDF) [file pone.0217472.s002.pdf]

S1 Table: Study Ethics Committees

Country/Site	Ethical Committee/MOH	Approval Date	Approval Ref.
Israel	MoH Israel	18.6.2007	HTA3740
Tel Aviv Sourasky Medical Center	Tel Aviv Sourasky Medical Center Ethical Committee	20.11.2006	06-172
India			
Postgraduate Institute of Medical Education and Research (PGI)	Post Graduate Institute of Medical Education & Research – Chandigarh Ethics Committee	4.7.2006	Micro/2006/2606
Nizam's Institute of Medical Sciences	Nizam Institute of Medical Science Ethics Committee	9.5.2006	EC/NIMS/614/2006
Jahangir Hospital, Pune	Hirabai Cowasji Jehangir Medical research Institute Ethics Committee	2.4.2007	No reference provided by the committee
Maulana Azad Medical College & Associated G.B Pant, L.N and GNEC Hospitals, Delhi	Maulana Azad Medical College Ethics Committee	21.2.2007	N.F.501(134)/EC/06/MC(Aca.)/ 2520
National Institute of Mental Health and Neuro Sciences, Bangalore	NIMHNS Ethics Committee	24.10.07	RPA/096/07
Hungary	MoH Hungary	8.1.2007	
Aladar Petz County Teaching Hospital, Gyor<br>Istvan Szechenyi University, Gyor	Medical Research Council, Scientific and Research Ethics Committee (ETT TUKEB)	9.11.2006	76-1-35/2006

University of Debrecen	Regional and Institutional Ethics Committee of the Science Council of the University of Debrecen, Medical and Health Science Centre	26.6.2006	2504-2006
B.A.Z. County Hospital	Regional/Local Committee of Science and Research Ethics<br>Borsod-Abaúj-Zemplén and Heves County	15.8.2007	N/A

Germany			
Neurology and Neurochirurgy University Clinic Leipzig	Ethical Committee for Medicine, Leipzig University Faculty	21.9.2006	180/2006
University Clinic, Heidelberg	Ethical Committee Medicine Faculty Heidelberg	17.8.2006	MV-189/2006
Department of Neurology and Stroke Center, University Hospital Essen	Ethical Committee Medicine Faculty University Hospital Essen	18.2.2008	08-3614
Friedrich-Alexander-University Erlangen	Ethical Committee Medicine Faculty Friedrich-Alexander- University Erlangen	18.9.2006	3545
Albert-Ludwigs University of Freiburg	Albert-Ludwigs-University-Ethical Committee<br>Freiburg	12.2.2008	43/08
