# Supplementary material for: Implant for Augmentation of Cerebral Blood Flow Trial-1 (ImpACT-1). A single-arm feasibility study evaluating the safety and potential benefit of the Ischemic Stroke System for treatment of acute ischemic stroke
Source: PLoS One. 2019 Jul 3;14(7):e0217472. doi: 10.1371/journal.pone.0217472 (PMC6609146; doi:10.1371/journal.pone.0217472)
Supplement: S3 File — Complete study protocol and clinical investigation plan. (PDF) [file pone.0217472.s004.pdf]

.Clinical Investigation Plan

Multi Center Feasibility Pilot Study:

Safety and Feasibility Assessment of the NeuroPath™ IS System in Treatment of Acute Ischemic Stroke – A Pilot Study

Protocol Number: CLP1000450
Revision: B
Date: July 2006

Principal Investigator:

1. Dr. Dheeraj Khurana
2. Dr. Subash Kaul
3. Dr. Shirish Hastak

Hospital name:

1. Post Graduate Institute of Medical Education and Research Chandigarh, Chandigarh.
2. Nizam's Institute of Medical Research Punjagutta, Hyderabad.
3. Lilavati Hospital Research Center, Bandra Reclamation, Bandra (West), Mumbai.

Study Sponsor:

BrainsGate Ltd.

17 Ha'Tidhar st. P.O.B 2249

Ra'anana 43654 Israel.

AUTHORIZATION				
	**Name**	**Title**	**Signature**	**Date**
Issued by	Adar Shani	Clinical Manager		
Approved by	Irit Yaniv	VP Clinical Affairs	I. Yaniv	
Reviewed by	David Katz	Director Regulatory Affairs and QA		
Principal Investigator	Dr. Dheeraj Khurana	Department of Neurology		

AUTHORIZATION

	Name	Title	Signature	Date
Issued by	Adar Shani	Clinical Manager		
Approved by	Irit Yaniv	VP Clinical Affairs		
Reviewed by	David Katz	Director Regulatory Affairs and QA		
Principal Investigator	Dr. Subash Kaul	Department of Neurology		

AUTHORIZATION

	Name	Title	Signature	Date
Issued by	Adar Shani	Clinical Manager		
Approved by	Irit Yaniv	VP Clinical Affairs		
Reviewed by	David Katz	Director Regulatory Affairs and QA		
Principal Investigator	Dr. Shirish Hastak	Department of Neurology		

Table of Content:

1 INTRODUCTION ... 7
1.1 Clinical Trial Rationale ... 7
1.2 Augmenting Brain Perfusion ... 7
1.3 The Role of Stimulation of the Sphenopalatine Ganglion in Ischemic Stroke ... 8
1.4 Preclinical Testing ... 9
1.4.1 Animal Studies of SPG Stimulation ... 9
1.4.2 The Evaluation of Safety of SPG Stimulation in Animal Studies ... 11
1.5 Previous Clinical Experience ... 13
1.6 Laboratory Testing ... 14
2 DEVICE DESCRIPTION ... 14
2.1 NeuroPath™ Identification ... 14
2.2 Intended Use and Indication ... 15
2.3 NeuroPath ™ Description ... 16
2.3.1 The Transmitter ... 17
2.3.2 The Driver ... 17
2.3.3 The Controller ... 17
2.3.4 The Implantable Neural Stimulator (INS) ... 18
2.4 Instruction for Installation (Controller) ... 18
2.4.1 Necessary Training ... 18
2.5 The Surgical Kit ... 19
2.6 Principles of Operation ... 20
2.6.1 General ... 20
2.6.2 Implantation of the Implantable Neural Stimulator (INS) ... 20
2.6.3 Stimulation with the Neuropath™ ... 22
3 STUDY INFORMATION ... 24
3.1 Overview ... 24
3.2 Summary of Study Design ... 24
3.3 Study Design ... 25
3.4 Study Objectives ... 25
3.4.1 Primary ... 25
3.4.2 Secondary ... 25
3.5 Study Endpoints ... 25
3.5.1 Primary endpoint ... 25
3.5.2 Secondary endpoint ... 26
3.6 Study Duration and Timelines ... 26
4 ELIGIBILITY CRITERIA ... 26
4.1 Inclusion criteria ... 27
4.1.1 Exclusion Criteria ... 27
4.2 Patient Enrollment ... 28
4.3 Patient Identification ... 28
4.4 Variables Measured ... 29
4.5 Procedural Protocol ... 29

4.5.1 Screening and enrollment 29
4.5.2 Baseline measurements 30
4.5.3 Treatment Period 31
4.5.4 Follow Up Period 31
5 Adverse Events 32
5.1 Definitions 32
5.2 Adverse Events Reporting 33
6 Statistical Analysis 33
6.1 Rationale 33
6.2 Interim Analysis 34
6.3 Patient Incorporation Policy 34
6.4 Data Accountability 34
7 INVESTIGATION ADMINISTRATION 34
7.1 Study Monitoring 34
7.2 On-Site Visits 34
7.3 Case Report Forms 35
7.4 Review of Study Documents 35
7.5 Patient Confidentiality 36
7.6 Study Discontinuation 36
7.7 Protocol Amendments 36
7.8 Study Documentation and Data Management 36
7.9 Records Retention 37
8 APPENDICES 38
8.1 Appendix A – Study Flow Chart 38
8.2 Appendix B - Trial measurements schematic 39

1 INTRODUCTION

1.1 *Clinical Trial Rationale*

Stroke is a leading cause of disability, death and health care expenditure. It is the second most common cause of death worldwide, exceeded only by heart disease, and the third most common cause in the US.[1, 2] In the US there are an estimated over 700,000 strokes (500,000 are first attacks and 200,000 recurrent events) claiming more than 150,000 lives annually.[2-4]

Stroke is caused by either an occlusion (ischemia) or hemorrhage within the cerebral circulation. Ischemic stroke constitutes 83 to 90 % of stroke cases in western countries.[2, 10] Occlusion of the Middle Cerebral Artery (MCA) or its branches is the most common site of ischemia, or cerebral infarct, in the anterior circulation accounting for approximately 90% of infarcts and two thirds of all first strokes. Of MCA territory infarcts over 50% involve the superficial MCA territory (Alison Baird, MD, PhD, Anterior Circulation Stroke, eMedicine.com).

The functionally impaired region that surrounds the infarct core and is threatened by necrosis has been termed the ischemic penumbra. The penumbra, although physiologically impaired, is potentially salvageable tissue. In the early phase following stroke, there is prompt initial improvement in function as the pathologic processes including edema, hemorrhage, and blood pressure (BP) resolve. The time frame for recovery of function in these reversibly injured neurons is relatively short, accounting for improvement in the first several weeks. The later ongoing improvement in neurologic function occurs by a different set of mechanisms that allow structural and functional reorganization within the brain. The processes involved in this reorganization represent neuroplasticity[1] and may continue for many months. Restitution of partially damaged pathways and expansion of representational brain maps occur, implying recruitment of neurons not ordinarily involved in specific activity.

To date, intravenous recombinant tissue plasminogen activator (IV t-PA) and mechanical revascularization with the MERCI device are the only two approved interventional treatments for acute ischemic stroke. Both are primarily focused on reperfusion, i.e. aiming to restore artery patency and brain perfusion in order to reduce the volume of cerebral infarction which thereby reduces functional deficits. However, the use of those treatments is limited. In the US, only about 3% of ischemic stroke patients are treated with IV t-PA. The main determinant of eligibility for treatment is time since onset; the time window for IV t-PA is less than 3 hours from stroke onset and up to 8 hours for the use of the MERCI device. The main determinant of eligibility for treatment is time since onset; the time window for IV t-PA is less than 3 hours from stroke onset and up to 8 hours for the use of the MERCI device. Symptomatic hemorrhagic transformation of the infarction remains the primary concern with the administration of IV t-PA in the treatment of acute ischemic stroke; up to 5.2% incidence is reported, based on a meta-analysis of the post marketing open-label studies published by Glenn. [53]

1.2 *Augmenting Brain Perfusion*

An alternative therapeutic approach in ischemic stroke, whenever early recanalization cannot be achieved, is augmentation of brain perfusion through the collateral circulation. The cerebral collateral circulation refers to the subsidiary network of vascular channels that stabilize cerebral blood flow when principal conduits fail. The collateral circulation is a critical determinant of cerebral perfusion pressure

[1] Neuroplasticity is the ability of the nervous system to modify its structural and functional organization.

in acute cerebral ischemia as is caused by stroke.[24] The quality of the process of collateral recruitment depends on the caliber and patency of primary pathways that may rapidly compensate for decreased blood flow and the adequacy of secondary collateral routes. Experimental data on middle cerebral artery occlusion in rats demonstrates the temporal dependence of collateral development.[25] The hemodynamic effects of the collateral circulation may also be important in maintaining perfusion to penumbral regions.[26]

Additionally, perfusion itself may enhance angiogenesis, which may further stimulate collateral growth at the periphery of an ischemic region.[27] Although numerous theoretical arguments suggest a possible beneficial effect of augmenting brain perfusion by increasing collateral blood flow to the brain, current management of cerebrovascular disease has not focused on this as a therapeutic approach in stroke treatment.

Preliminary data suggest that induced hypertension in acute ischemic stroke may be associated with a reduction in hypoperfused tissue on perfusion-weighted MR scans, and may potentially improve outcome.[28-30]. The mechanism of improved function is assumed to be the enhanced cerebral perfusion.

There remains a pressing need for developing additional therapies for the treatment of acute ischemic stroke at a longer therapeutic window as well as for an improvement in outcome during the rehabilitation phase. For the acute phase, the therapeutic focus is in limiting the ischemic insult by restoration or alleviation of the Cerebral Blood Flow (CBF). Other interventions, referring to a time point much later after onset of stroke, are intended to help in the rehabilitation process and in particular to intervene in the process of brain plasticity. The mechanism of improved function is assumed to be the enhanced cerebral perfusion. The two most plausible forms of plasticity are collateral sprouting of new synaptic connections and unmasking of previously latent functional pathways. This would be clinically presented as functional improvements. Experimental evidence indicates that plasticity can be altered by several external conditions, including pharmacologic agents, electrical stimulation, and environmental stimulation.

1.3 *The Role of Stimulation of the Sphenopalatine Ganglion in Ischemic Stroke*

The sphenopalatine ganglion (SPG), classically known to be the source of parasympathetic innervations to the nasal and eye mucosa and the lacrimal gland, is now recognized also as the source of parasympathetic innervations to the brain vasculature. The location of the SPG is shown in Figure 1.3-1.

Figure 1.3-1- Anatomical Location of Sphenopalatine Ganglion (SPG)

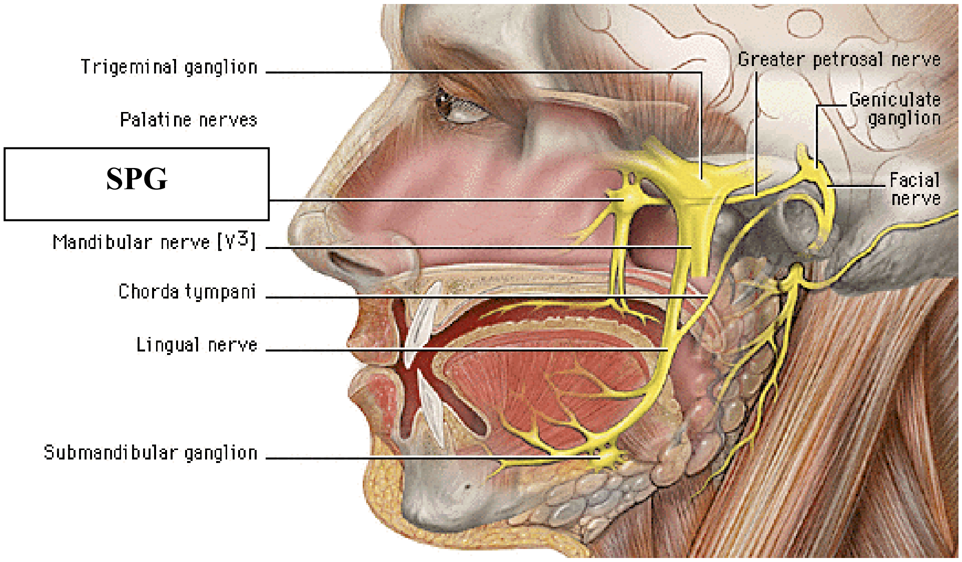

Stimulation of this ganglion induces vasodilatation of the ipsilateral, intradural arteries of the anterior circle of Willis in normal rats, cats, dogs, monkeys and human. The neurotransmitters released by the postganglionic fibers include nitric oxide (NO), vasoactive intestinal polypeptide (VIP), and acetylcholine, as well as other peptides of the VIP family such as peptide histidine isoleucine and its human form, peptide histidine methionine.[38-43] NO and VIP are known vasodilators of cerebral arteries *in vitro*. Vascular dilation *in vitro* and increases in cerebral blood flow (CBF) *in vivo* in response to SPG stimulation are blocked by antagonists of NO and VIP antiserum. There appears to be sufficient evidence in the literature which supports coupling between NO, vascular endothelial growth factor (VEGF) and angiogenesis, indicating that NO is involved in the regulation of progenitor cells and neurogenesis in the adult brain. Administration of NO in animal stroke models was shown to increase CBF to the ischemic territory, reduce the tissue damage resulting from focal Ischemia and enhance neurogenesis. [54]

1.4 *Preclinical Testing*

1.4.1 Animal Studies of SPG Stimulation

Substantial experimental evidence exists to indicate that as a result of SPG stimulation, nitric oxide (NO) and vasoactive intestinal peptide (VIP) are released from the nerve ending of the ganglion to accelerate, via specific receptors, vasodilatation of cerebral vessels. Regulation of the latter physiological phenomenon depends upon, among other parameters, the availability of neurotransmitters and neuromodulators and their rate of clearance at the site of action. A well-established electrical pulse regimen of the SPG is believed to be essential for controlling the magnitude and duration of

vasodilatation, enhance cerebral perfusion and consequently improve neuromotor and cognitive functions.

It was previously demonstrated, that the duration and magnitude of the pulse provided by the BrainsGate System using the NeuroPath™ IS System electrode may differentially affect neuronal networks that are activated down stream by the SPG stimulation.

BrainsGate has completed a vast amount of pre-clinical studies to demonstrate the effects of SPG stimulation in animals with cerebral ischemia. The stroke model utilized by BrainsGate consists of an occlusion of the Middle Cerebral Artery (MCAo), a commonly used model for generating cerebral ischemic stroke in rats. Functional impairment has been attained by a transient (tMCAo) or a permanent (pMCAo) occlusion resulting into neurological deficits and long-term or permanent motor dysfunction. The consequence of either mode of occlusion has been assessed in terms of infarct size, and neurological and cognitive improvement using verified scoring systems.

Studies were aimed at specifying and optimizing the electrical pulse regimen for SPG stimulation as well as the treatment duration. Specific aims included:

- Identification of the appropriate stimulation profile for the stroke application.
- Assessment of short and long term effects as measured by cerebral blood flow (CBF), infarct size and neurological improvement; follow up duration lasted up to 35 days.
- Evaluation of the therapeutic window following stroke onset; from immediate stimulation post stroke up to treatment 24 hours post stroke.
- Selection of suitable treatment regimen (i.e. treatment duration, and number of stimulations per day).

Table 1.4-1 below provides an overview of the pre-clinical animal studies which were completed during the development of the NeuroPath™ IS System. In summary, results from completed studies indicate that:

- The [60”/12”] stimulation profile is the most suitable paradigm for further research in ischemic stroke.
- Short term efficacy is demonstrated after one 5 min train of SPG stimulation, and following long lasting stimulation (up to 10 h). Evaluation of efficacy parameters showed an increase in CBF, a decrease in infarct volume and improvement in neurological function as measured by a series of behavioral tests detailed in the literature.
- Improvement of the above parameters can be shown after extended delays (3 h and 24 h) following occlusion.
- Repetitive SPG stimulation attenuated neurological deficit and reduced infarct size after 24 h following occlusion.
- Enhanced cognitive and neurological functions were found in both tMACo and pMACo animals as a result of SPG stimulation.

- Beneficial effects can be observed up to 4 weeks after the MCA occlusion, and may be attributed to either the long-term influence of SPG stimulation, or to a long-term rehabilitation process.
- The most efficient treatment duration was demonstrated when the treatment lasted for 3 or 6 h. When the stimulation period increased to 10 h or decreased to 1 h, the beneficial effects diminished.

Table 1.4-1 – Summary of Animal Studies of SPG Stimulation

**Study Title**	**Study Number**	**Results**
Effect of sphenopalatine ganglion (SPG) stimulation on cerebral blood flow (CBF) in non-ischemic rats	BG27	Results support the [60”/12”] stimulation as the most suitable pulse protocol. The effect is long lasting, indicative of sustained vasodilatation
Effect of SPG stimulation on ischemic efficacy parameters	BG34	SPG stimulation (5 minutes) followed by a relaxation period of 25 minutes repeated over 10 hours, markedly increased CBF levels; substantially decreased the infarct volume; and significantly improved the neurological function. Effects were recorded at 3 h and 24 h following occlusion.
Modulation of ischemic parameters by prolonged SPG stimulation	BG35	SPG stimulation had an effect on CBF up to 48 h following SPG stimulation; infarct size was reduced.
Repetitive SPG stimulation and its impact on ischemic parameters	BG10	Three consecutive sessions of SPG stimuli were effective in increasing the neurological scoring outcome and substantially reducing the size of the ischemic infarct.
The effect of SPG stimulation (24 h delay after stress) on tMCAo model	BG25	SPG stimulation following transient MCAo is a suitable preparation to study the long-term consequences of ischemic stroke. The study demonstrated a clear and positive effect of SPG stimulation with respect to enhanced cognitive and neurological functions.
The effects of SPG stimulation for different treatment duration (24 h delay after stress) on tMCAo model; follow up period up to 35 days	BG41	Several behavioral and cognitive tests were performed; an overall analysis was done for the results obtained at 35 days follow up. It was clearly demonstrated that the best response could be demonstrated in the 3 or 6 hours SPG stimulated groups.

1.4.2 The Evaluation of Safety of SPG Stimulation in Animal Studies

BrainsGate performed several studies in animals to evaluate specific parameters and aspects of the safety of SPG stimulation. These studies are identified in Table 1.4-2.

Table 1.4-2 – Summary of Safety Studies

**Study Title**	**Study Number**	**Results**
SPG stimulation and general pathophysiological criteria	SPG-S1	SPG stimulation did not cause any visible pathological signs or neurological adverse side effects
Effects of SPG stimulation on brain edema	SPG-S2	There were no observations of cerebral edema or edema-related adverse side effects following short term or long term SPG stimulation.
Effects of SPG stimulation on cell death by apoptosis	SPG-S3	No cell damage was observed 24h after SPG stimulation under conditions known to increase delivery of molecular markers or drugs into the brain.
Acute SPG stimulation and motor and cognition activity	SPG-S4	Following acute and single SPG stimulation there were no significant changes in body weight, clinical signs, motor function or any unusual findings in the open field test.
Subchronic SPG stimulation and motor activity and cognitive behavior	SPG-S5	Adverse effects in behavior were observed and reversed by 21 days post-stimulation. These effects may relate to a generalized stress/anxiety behavior induced by repeated SPG stimulations. Noted effects are consistent with well documented adverse side effect as a result of repetitive stressing in rodents
Chronic SPG stimulation and motor activity and cognitive behavior	SPG-S6	Minor reversible adverse side effects were observed, predominately in the 3h stimulation group. These adverse effects (changes in body weight and in the open field) are similar to the changes observed during the sub-chronic stimulation experiment and could be explained by the procedure, i.e., related stress as a result of repeated SPG stimulations.

In summary, through the use of well-defined animal models for ischemic stroke, BrainsGate has demonstrated that cerebral blood flow is increased as a result of SPG stimulation. Additionally, in animals with permanent occlusion of the middle cerebral artery, SPG stimulation appears to be associated with a small increase in recovery of neurological function, and a substantial reduction of brain infarct volume. In animals with transient ischemia, there was a substantial recovery of motor and cognitive abilities as a result of SPG stimulation.

Additionally, the safety studies demonstrate no long-term adverse events as a result of SPG stimulation at stimulation levels (i.e. duration, stimulation on/off times, and current levels) in excess of those utilized to demonstrate efficacy in the ischemia stroke model. Short term effects on weight gain, open field tests, and cognitive ability were observed in SPG stimulated rats; all effects were reversible within 9 weeks after the termination of stimulation. Furthermore, there were no histopathologic findings of an adverse nature.

Based on these studies, it is concluded that SPG stimulation may have a therapeutic role in the treatment of patients following ischemic stroke, supported by a positive benefit to risk assessment.

1.5 *Previous Clinical Experience*

The first clinical studies utilizing BrainsGate technology were initiated in Europe in 2004. These Studies investigated the device operation using different stimulation regimen, as a controlled method for destruction the Blood-Brain-Barrier by stimulating the SPG.

In these Pilot feasibility studies, the Neuropath™ System was used to improve the bioavailability of the chemotherapeutic agent, Paclitaxel, which normally does not penetrate the CNS. The study population was Glioblastoma Multiform (GBM) patients with histological evidence of GBM. In these patients, disease recurrence was documented, and the tumor was inoperable. The primary objective of the study was to assess safety of the device implantation procedure and the stimulation protocol. Efficacy parameters including the increase in cerebral blood flow, drug level contained in the cerebral spinal fluid (CSF) and S100 protein were established as secondary objectives. In addition, follow-up on tumor response was performed.

To date, the NeuroPath™ System has been implanted in ten (10) GBM patients in Europe: six in Hungary, three in Croatia and one in Germany.

Clinical data obtained from these subjects includes:

- The clinical response to SPG stimulation (i.e. tolerance to stimulation);
- Safety parameters including anticipated and unanticipated adverse events; related and unrelated to the device and the procedure;
- And, efficacy parameters as specified above;

In summary, the implantation procedure in patients with GBM, followed by the specific protocol of SPG electrical stimulation, was shown to be safe and tolerable. During the stimulation protocol, only minor symptoms were observed on the stimulated side of the head. As mentioned above, those symptoms are expected while the SPG is stimulated. Moreover, BrainsGate and its Clinical Investigators view the appearance of these minor symptoms as positive indicators of a successful SPG stimulation session. During the clinical use of the NeuroPath™ System in patients with GBM, there have been no device- related serious adverse events.

Feasibility for the treatment of GBM was further demonstrated quantitatively by evidence of cerebral vasodilatation, elevation in S100 protein and elevation in drug levels in CSF, all indicators of enhanced drug delivery across the blood brain barrier.

These results, coupled with the findings of minimal adverse events, suggest that SPG stimulation possesses clinical utility and may be used under controlled conditions in a clinical study involving subjects impaired by ischemic stroke. Furthermore, with implementation of device and procedural improvements, BrainsGate believes that the device-related adverse events experienced in these early clinical trials for GBM may be further mitigated.

Risk analysis can be found in the Investigator Brochure.

1.6 *Laboratory Testing*

The NeuroPath™ IS System was tested for compliance with EN 60601 electrical safety & EMC standards as well as sent for biocompatibility testing (NAMSA laboratories). No safety concerns were raised. Refer to the Investigator Brochure for details.

2 DEVICE DESCRIPTION

2.1 *NeuroPath™ Identification*

Manufacturer:	BrainsGate
Device:	NeuroPath IS System
Device components:	Implantable Neural Stimulator Model 400 - P/N FP2-000401, FP2-000451
Controller Model 400-	P/N FP3-000405
Driver Model 400-	P/N FP3-000404
Transmitter Model 400-	P/N FP3-000402

Surgical Accessories supplied

Custom accessories to be provided:

- 1.00 mm rounded trocar 10°
- 1.10 mm rounded trocar 10°
- 1.20 mm rounded trocar 10°
- 1.30 mm rounded trocar 10°
- 1.40 mm rounded trocar 10°
- 1.50 mm rounded trocar 10°
- 1.60 mm rounded trocar 10°
- 1.70 mm rounded trocar 10°
- 1.80 mm rounded trocar 10°
- 1.90 mm rounded trocar 10°
- 2.00 mm rounded trocar 10°
- Stylet
- Palatometer

Standard accessories provided:

- Goldman Fox scissors
- Obwegesser periostreal elevator
- Standard perosteal elevator #9
- Jeter pharyngeal or De Bakey needle holder
- Edentulous black mouth prop
- Adult black mouth prop
- Micro Adson tissue and Anatomical Adson forceps
- Scalpel handle #3
- Davis-Boyle mouth gag with 60x22, 75x25, 90x25 and 107x27mm blades
- Dental aspiration syringe
- Forceps Foerster-Ballenger 25cm
- Suction Younkauer 27 cm
- Small bowl 40 mm X 19 MM
- Small bowl 80 mm X 40 MM
- Lucas curette
- Horigan periosteal elevevator
- Buser surgical chisel

2.2 *Intended Use and Indication*

The NeuroPath™ IS System is intended for electrical stimulation of the Spheno-Palatine Ganglion (SPG) to augment cerebral perfusion and aid in the management of ischemic cerebral stroke, i.e. improving neurological deficits, of ischemic stroke patients, who are not eligible for reperfusion therapy.

The system is designed to enable repetitive continuous sessions of electrical stimulation of the sphenopalatine ganglion (SPG) for 7 days following acute ischemic stroke. It is assumed, based on the literature and the BrainsGate pre clinical work, that usage of the system and the treatment regimen is safe, and tolerable.

Patients receiving the stimulation treatment are expected to show an improvement in their ischemic stroke status. The improvement is expected to be demonstrated by the augmentation of cerebral blood flow during and immediately after stimulation; reduction of the lesion measures and consequently improvement of the neurological deficits. As such, the treatment with the device may therefore provide a solution for acute ischemic stroke patients, who cannot benefit from early recanalization therapies.

The suggested hypothetical mechanism of action discusses the option that the enhancement of endogenous neurogenesis via enhanced release of NO and VIP, occurs due to better perfusion status; this sequentially boosts the physiological process of brain plasticity and results in improvement in the functional deficits despite artery occlusion.

The NeuroPath IS System provides an opportunity of stimulation during the penumbra phase and later in the early sub acute phase to augment cerebral perfusion. Resulting in the opportunity to

provide a simple, safe, minimally invasive approach for long-term delivery of NO and VIP to the ischemic area.

2.3 *NeuroPath ™ Description*

The NeuroPath™ IS System is comprised of two major components:

The Implantable Neural Stimulator (INS)

The Energy Delivery Control Subsystem (EDC).

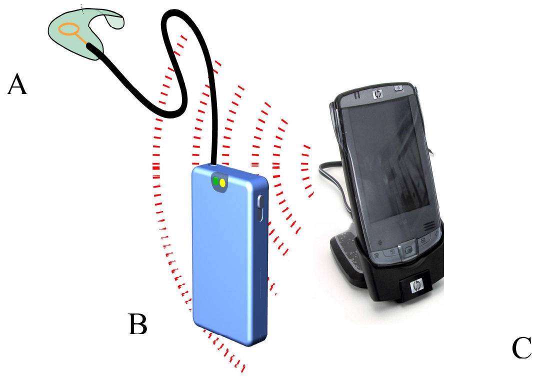

A	The Transmitter
B	The Driver
C	The Controller

The INS is implanted in the vicinity of the SPG via the greater palatine canal; the EDC controls the stimulation by way of energy delivered to the INS via electromagnetic coupling.

The EDC is further divided into three parts:

- The Transmitter
- The Driver
- The Controller

A surgical kit provided with the NeuroPath™ IS System, facilitates the INS implantation procedure.

2.3.1 The Transmitter

The Transmitter is used to relay energy between the EDC Subsystem and the Implantable Neural Stimulator (INS). The Transmitter is a single-patient reusable device component that requires cleaning before each use. The Transmitter is mechanically held in place facing the INS receiver inside the oral cavity.

2.3.2 The Driver

The Driver is the interface between the Controller and the Transmitter. It is physically connected to the Transmitter and communicates with the Controller in a wireless fashion. This communication consists first of loading with the stimulation parameters by the physician/healthcare provider, followed by exchanging information and data during treatment. The Driver is reusable.

2.3.3 The Controller

The Controller, an off-the-shelf, hand-held computer, fitted with custom Microsoft© Windows based software, controls the stimulation pattern and the stimulation timing, and collects and archives the log files of the treatment sessions. During the operational phase, the Controller communicates with the Driver via a Bluetooth© communication protocol for the purpose of beginning a treatment session, verifying correct operation and logging data for the treatment sessions. The Controller is powered by an internal battery that is charged by a standard AC medical grade power supply that is supplied with the system. The Controller is reusable.

2.3.3-1: The Controller

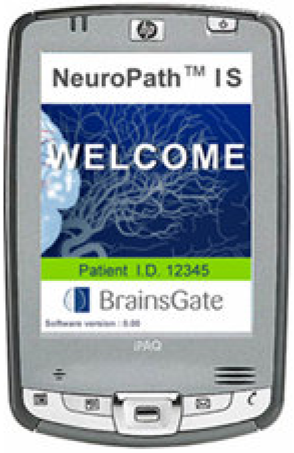

2.3.4 The Implantable Neural Stimulator (INS)

The INS is composed of three major areas (see Figure 2.3.4-1):

1. The receiver (electrical circuit) ("A" on Figure 2.3.4-1)
2. The body of the INS ("B" on Figure 2.3.4-1)
3. The INS contact tip for stimulation ("C" on Figure 2.3.4-1)

For full description of the Implantable Neural Stimulator (INS) structure please refer to the investigator brochure

Figure 2.3.4-1: The Implantable Neural Stimulator (INS) – model 400

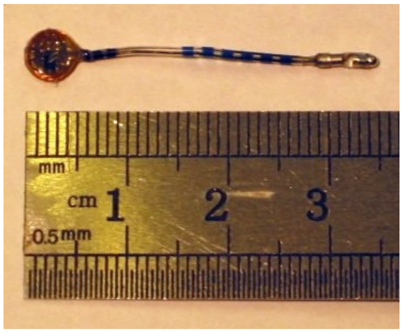

2.4 *Instruction for Installation (Controller)*

The stimulating controller does not need to be installed. For complete instructions on the operation of the NeuroPath™ IS System refer to the NeuroPath™ IS System Physician Manual.

2.4.1 Necessary Training

The usage of the NeuroPath™ **IS** System is composed of two processes:

1. The implantation procedure.
2. The stimulation procedure.

In this pilot study, the insertion procedure will be performed by the local surgeons following a training session, and under the supervision of a BrainsGate surgeon who is trained and experienced in the implementation surgical procedure.

The stimulation procedure requires a trained person to be present during the session. During the first stimulation procedures BrainsGate representative will be present, and will assist the investigator with the operation of the NeuroPath™ IS System.

2.5 *The Surgical Kit*

The surgical accessory kit is composed of custom made tools intended for use in the preparation of the greater palatine canal for insertion of the Implantable Neural Stimulator (INS). Other tools supplied are conventional surgical tools intended to facilitate exposure of the greater palatine canal and aid in the overall procedure.

The custom-made tools supplied as part of the surgical kit consist of:

1. A set of rounded trockars ranging in diameter from 1.00 to 2.00 mm (in 0.1 mm increments), with a tip angle of 10.°
2. A stylet with a finely sharpened tip (1.3 mm in diameter) for initial penetration into the greater palatine canal.
3. A palatometer, dedicated tool used to locate the entrance of the greater palatine canal.

The conventional surgical tools supplied consist of:

1. One Goldman Fox scissors
2. One Obwegesser periosteal elevator
3. One standard periosteal elevator #9
4. One Jeter pharyngeal or De Bakey needleholder
5. One Edentulous black mouth prop
6. One adult black mouth prop
7. One Micro - Adson tissue forceps
8. Two Adson dressing forceps
9. One scalpel handle #3
10. One mouth gag, including four blade sizes: (107x27, 90x25, 75x25 & 60x22 mm).
11. One dental aspiration syringe
12. One forceps 25 cm
13. One Younkauer suction 27 cm
14. One small bowl 40 x 19 mm
15. One small bowl 80 x 40 mm
16. One Lucas curette
17. One Horigan periosteal elevator
18. One surgical chisel

2.6 *Principles of Operation*

2.6.1 General

The system is designed to enable repetitive continuous sessions of electrical stimulation of the SPG for 7 days following acute ischemic stroke event. Patients receiving the stimulation treatment are expected to show an improvement in their ischemic stroke status. The improvement is expected to be demonstrated by the augmentation of cerebral blood flow during and immediately after stimulation. Reduction of the lesion measures and consequently improvement of the neurological deficits are expected to be demonstrated later on during the follow up period. .

The suggested hypothetical mechanism of action discuss the option that the enhancement of endogenous neurogensis via enhanced release of NO and VIP, occurs due to better perfusion status; this sequentially boosts the physiological process of brain plasticity resulting in improvement in the functional deficits despite artery occlusion.

2.6.2 Implantation of the Implantable Neural Stimulator (INS)

The implantation of the INS in the treatment of ischemic stroke is intended to be performed by a surgen such as Otolaryngologists, Maxillofacial Surgeons or Neurosurgeons who will receive specialized training by BrainsGate. The participating surgeons will be trained to perform the procedure on cadavers before attempting in a clinical setting. Implantation of the INS takes approximately 20 minutes to complete.

The following section provides an overview of the surgical procedure for the implantable component (INS) of the Neuropath™ IS System.

1. The patient is locally anesthetized.
2. The entrance to the Greater Palatine canal is located using standard procedures well known to dentists which routinely deliver anesthesia injections into the canal.
3. An incision is made adjacent to the canal entrance within 5 mm.
4. The canal is exposed, and a path is created alongside the canal with the aid of surgical tools.
5. The INS is surgically inserted via the greater palatine canal, such that the stimulating tip rests adjacent to the SPG and its receiving end is situated underneath the mucosa of the Palatine process of the maxilla bone or potentially of the Alveolar process of the maxilla bone. Positioning is obtained by introducing the INS until the distal stimulating tip reaches the superior boundary of the Sphenopalatine fossa.
6. The incision is sutured or glued.
7. The Implantable Neural Stimulator (INS) will be removed at the end of the treatment portion of the study, i.e. between 7 - 28 days after implantation.

Figure 2.7.2-1 shows an X-Ray image of the Implantable Neural Stimulator (INS) as it appears in a Glioblastoma Multiforme patient post implantation.

Figure 2.7.2-1 – X Ray image of an implanted Implantable Neural Stimulator

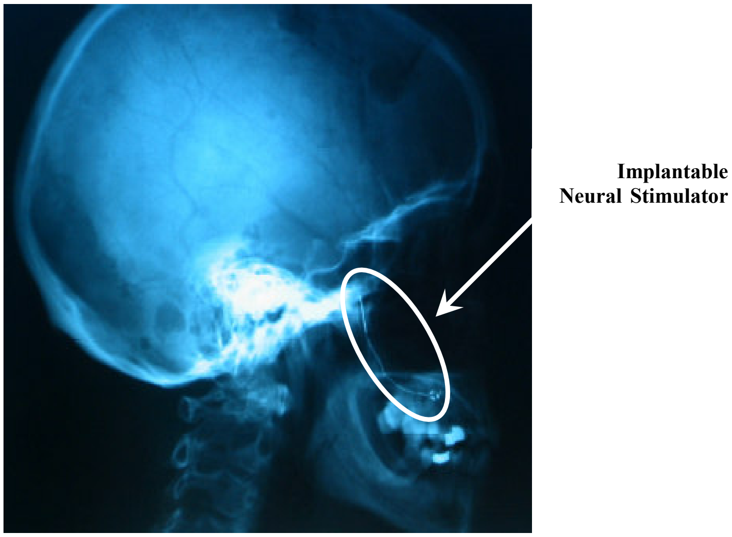

2.6.3 Stimulation with the Neuropath™

Immediately (up to 15 minutes) following implantation, the Transmitter is placed in the patient's mouth such that it is aligned with the Implantable Neural Stimulator (INS) receiver. There are predefined stimulation parameters which are set in the Controller during manufacturing (frequency, stimulation times etc). Then, there are stimulating parameters which could be set in the Controller by the Physician (pulse width, stimulation intensity). In any case, the patient has no control over the setting of the stimulation parameters. Upon activation, the Controller transmits the stimulation parameters to the Adaptor. The energy for the stimulation is driven by an energy source and electronic driver within the Adaptor. The Adaptor conveys the energy via a linear connection cable to the Transmitter. Finally, the Transmitter then transmits the energy to the Implantable Neural Stimulator (INS), electrically stimulating the SPG. Stimulus strength will be administered at an approximate range of 2 mA ± 1.

The rationale for selecting the stimulation parameters of the NeuroPath™ IS System is based upon two primary sources. [2,3] Both address the parameters of nerve stimulators, although neither with respect to the CNS. These standards are considered to be the closest relevant to the NeuroPath™ IS System. Listed below is a summary of the key safety and performance requirements of each.

- ANSI/AAMI NS15-1995 Implantable Peripheral Nerve Stimulators – Stimulation Parameters
  a. Pulse frequency: 1 to 1,500 pulses per second (pps).
  b. Pulse width: 1 to 2,000 microseconds (µsec).
  c. Output voltage/current: 0 to 15 volts (V) through a 500 ohm load (0 to 30 mA).
  d. Waveform: Shall consist of balanced positive and negative phases, so that the net dc current through the electrodes does not exceed 10µA.
- ANSI/AAMI NS4-1985 Transcutaneous Electrical Nerve Stimulators – Safety Considerations
  a. Maximum charge per pulse: 75 microcoulombs (µC) into a 500 ohm load.
  b. Maximum average current: 10 milliamperes (mA) into a 500 ohm load.

---

2 The American National Standards Institute (ANSI) / The Association for the Advancement of Medical Instrumentation (AAMI) NS15-1995 Implantable peripheral nerve stimulators.

3 The American National Standards Institute (ANSI) / The Association for the Advancement of Medical Instrumentation (AAMI) NS4-1985 Transcutaneous electrical nerve stimulators.

Table 2.7.3-1- NeuroPath™ IS System Characteristics

**Characteristic**	**NeuroPath™ IS System (at 500 ohm)**
Voltage (V)	0 - 2
Current (mA)	0 - 4
Pulse width (µsec)	60 - 1000
Frequency (pps)	10 - 30
Charge per pulse (µC) at max pulse width	3
Materials<br>7.1. Electrode<br>7.2. Insulator<br>7.3. Other	1. Platinum/Iridium<br>2. Polycarbonate urethane & silicone rubber<br>3. Epoxy
Design features	• Single electrode<br>• Cylindrical
Sterilization	EtO

3 STUDY INFORMATION

3.1 *Overview*

In this pilot feasibility study, the NeuroPath™ IS System will be used to assess the feasibility and safety during treatment initiated up to 24 hours following acute ischemic stroke in a comparison method.

The study population will be acute ischemic patients. The system is designed to enable repetitive continuous sessions of electrical stimulation of the sphenopalatine ganglion (SPG) for 7 days following acute ischemic stroke.

A total of 30 (thirty) patients with acute ischemic stroke (within the last 24 hours) will be enrolled to this study. The patients who will be enrolled to the treatment group will undergo an implantation procedure in which the NeuroPath™ IS System will be inserted into the SPG by using a minimal invasive approach; thereafter the stimulation treatments will begin and will continue 7 days. The patient should stay in the hospital for the whole treatment duration. The patients who will be enrolled to the control group will receive a standard medical care without a stimulation procedure.

A follow-up session will take place for up to 90 days post enrollment to the trial to both groups.

The entire process is expected to result in an improvement in the patient's neurological deficits and functional status after the ischemic stroke.

3.2 *Summary of Study Design*

Hypothesis:	The use of the NeuroPath™ IS System is safe and tolerable when use for cerebral ischemia treatment.<br>The use of the NeuroPath™ IS System will augment cerebral perfusion and aid in the management of ischemic cerebral stroke, i.e. improving neurological deficits of ischemic stroke patients.
Design:	A Pilot, prospective, comparison, multi center, study
No. of Centers:	1. Post Graduate Institute of Medical Education and Research Chandigarh, Chandigarh.<br>2. Nizam's Institute of Medical Research Punjagutta, Hyderabad.<br>3. Lilavati Hospital Research Center, Bandra Reclamation, Bandra (West), Mumbai.
Study Group	15 patients will undergo NeuroPath™ IS System implantation and a stimulation procedure.
Control Group	15 patients will receive standard of care without a stimulation

	procedure.
No. of Subjects:	A total of 30 subjects will participant the trial and will be compared in ration 1:1.
Study duration and Follow-up:	The overall length of treatment will be 7 days, with daily stimulation sessions of 3 hours duration.<br>Follow-up will be performed at 30±5, at 60±5 and at 90±5.
Endpoints:	1. Rate of intra-procedural and post-procedural adverse events.<br>2. Improvement of cerebral perfusion during and immedialty after stimulation<br>3. Improvement of neurological scale performed to ischemic stroke patients at different time points.

3.3 *Study Design*

This is a pilot study designed as an open label, comparison, multi-center study. The primary objective of this study is to demonstrate feasibility and safety of the device and the procedure**.**

The treatment group will be treated with the NeuroPath IS System as an adjunct to conventional non thrombolytics medical care.

The control group will be treated with a standard medical care.

3.4 *Study Objectives*

3.4.1 Primary

The objective of this pilot study is the assessment of the feasibility and the safety of the NeuroPath IS System in patients who have experienced an acute ischemic stroke in the MCA territory.

3.4.2 Secondary

The secondary study's outcomes are related to the patient's status from the ischemic stroke.

These outcomes measures include the following:

1. Augmentation of perfusion and/or cerebral blood flow changes
2. Infract size/volume changes
3. Changes in the neurological and functional status.

3.5 *Study Endpoints*

3.5.1 Primary endpoint

Those endpoints will include the assessment of the safety and feasibility of the device activation.

For the safety analysis: the incidence of the procedure complications, Expected/Unexpected Adverse Events and SAE will be recorded

For the feasibility analysis: the information will be collected. Patient's tolerability of the stimulation will be assessed. The outcome will be measured as the ability to maintain treatment in each session for the treatment length, and the compliance for the whole treatment period; i.e. each treatment session should be performed for at least 85% of the prescribed regimen and the compliance of the treatment should reach at least 85%. Reports will include questionnaires specifically designed to evaluate tolerance; and data regarding treatment compliance.

3.5.2 Secondary endpoint

Those are related to cerebral brain perfusion and the patient's status from the ischemic stroke. These outcomes measures include:

A. cerebral perfusion (MRI perfusion/ CT perfusion and SPECT scan);

B. cerebral blood flow velocity measured at the implant side (TCD);

C. infract size/volume changes;

D. and the changes in the neurological and functional status.

3.6 *Study Duration and Timelines*

The study will be performed in two (2) sites with the goal of enrolling 30 patients. Our estimation is that the site will enroll 4 patients per month with a total enrollment period of 4 months. The trial will involve an implantation of the NeuroPath™ IS System in 15 patients (treatment group), thereafter the patients will receive treatments for a duration of 7 days, with daily stimulation sessions of 3 hours duration. The additional 15 patients (control group) will receive a standard medical care without a stimulation procedure.

Follow-up will be performed to both groups at 30±5, 60±5 and at 90±5; aggregate trial duration will be ~100 days per patient.

Database cleaning and lock will take one month and final statistical analysis and report additional two months.

4 ELIGIBILITY CRITERIA

30 Patients with acute ischemic stroke (within the last 24 hours) who will meet the inclusion/exclusion criteria will be assigned by the physician in charge of the study ("Principal Investigator") to one of two groups: either a group that receives a treatment with the NeuroPath IS System or a group that does not receive a treatment with the NeuroPath IS System. (If patient does not fulfills Exclusion criteria #21 but the other criteria's are met, only than the patient will be assigned to the control group).

4.1 *Inclusion criteria*

All of the following must be answered YES in order to enroll a patient to this study:

1. Age: ≥ 18 years and ≤ 75 of both gender
2. Clinically diagnosed hemispheric cortical infarction, with in the MCA territory with clinical signs of cortical involvement
3. NIHSS ≥ 7 and ≤ 16
4. NIHSS – motor score (leg plus arm) equal or greater than 4
5. Treatment can be initiated within the first 24 hours following stroke onset
6. Signed informed consent has been obtained from the patient him/herself or his/her legally authorized representative

4.1.1 Exclusion Criteria

Any one of the following will exclude the patient from participation in this study:

1. Time interval prior to treatment initiation in greater than twenty-four (24) hours
2. Time interval since onset of symptoms undetermined.
3. Treatment with NeuroPath^{TM} IS System can't start within the first 24 hours post stroke onset
4. Parenchymatous Hemorrhage on imaging
5. Any other imaging diagnosis including tumor, abscess, intracranial hemorrhage (ICH); or symptoms suspicious for subarachnoid hemorrhage, etc.(on MRI or CT - significant mass effect, edema, or midline shift including Screening DWI abnormality greater than 2/3 of the MCA territory.)
6. Strictly subcortical infarct (i.e. no cortical involvement)
7. Not a MCA territory stroke
8. Minor stroke with non-disabling deficit or rapidly improving neurological symptoms with a high probably to Transient Ischemic Attack (TIA)
9. Eligible to or treated with IV or IA t-PA or mechanical thrombolysis
10. Baseline NIHSS greater than 16 or lesser than 7
11. Neurological deficit that has led to stupor or coma (NIHSS level of consciousness score greater than or equal to 2)
12. History of stroke in previous 6 months
13. Previously disability; Modified Rankin Score greater than 2
14. Patients under oral anticoagulants or having received heparin within 48 hours, and / or with elevated activated partial thromboplastin time (aPTT) (or INR)
15. High clinical suspicion of septic embolus

16. Severe cardiac disease: evidence of congestive heart failure or has history of end-stage cardiovascular disease (e.g. CHF NYHA Class III or IV or unstable angina)
17. Uncontrolled hypertension (systolic >185 mmHg and/or diastolic >110 mmHg)
18. Serious systemic infection
19. Women known to be pregnant, lactating or having a positive or indeterminate pregnancy test
20. Patients with other implanted neural stimulator
21. Orthodontics or non-Hygienic condition/ problems that prevent procedures within the mouth (minimum of TBD teeth on the stimulated side are necessary)
22. MRI[4] contraindications, such as but not limited to:
    - ✓ *Central nervous system aneurysm clips*
    - ✓ *Implanted cardiac pacemaker or defibrillator;*
    - ✓ *Cochlear implant*
    - ✓ *Ocular foreign body (e.g. metal shavings)*
    - ✓ *Insulin pump*
    - ✓ *Metal shrapnel or bullet*
    - ✓ *Any implanted device that is incompatible with MRI.*
    - ✓ *Patients with a condition precluding entry into the scanner (e.g. morbid obesity, claustrophobia, etc.)*
23. Life expectancy < 1 year from other causes
24. Currently participating in any other clinical trial
25. Patients unable or unwilling to follow protocol requirements

4.2 *Patient Enrollment*

Informed Consent will be obtained and then eligibility screening will be assessed using Inclusion/ Exclusion criteria.

A patient who appears to meet **ALL** eligibility requirements will be asked to participate in the study.

4.3 *Patient Identification*

The patient identification number will be of 4 characters; the first two characters correspond to the site number, and the last three characters correspond to the patient number at the site, as follows:

- Site number will be composed of two numbers; "01"
- The patient CRF number will be composed of one character; "001", "002" etc.

[4] This exclusion criteria may be disregarded if CT scan will be used as the imaging modality.

4.4 *Variables Measured*

The primary end point will be evaluated following and during each activation session.

- **Expected/Unexpected Adverse Events and SAE** will be evaluated during and immediately following the activation, as well as self reported (or otherwise obtained) absence of side effects between stimulations will be considered as a positive primary end point.
- **Patient's tolerability of the stimulation** will be assessed. The outcome will be measured as the ability to maintain treatment in each session for the treatment length, and the compliance for the whole treatment period

The secondary end points will be measured as follows:

- **Cerebral Perfusion** - Cerebral perfusion changes as measured by using imaging modalities (MRI/CT perfusion and/or SPECT) scan resulted from the stimulation. Recording will be performed for all treated patients, in a sample of the treatment sessions. It is required to performed at least 2 measures – during the first day of treatment and the last day of treatment.
- **Cerebral Blood Flow** - Cerebral blood flow velocity changes as measured by Transcranial Doppler (TCD) sonography resulted from the stimulation. Recording will be performed for all treated patients, in a sample of the treatment sessions. Recording will include the time before, during and following the stimulation session and will be compared.
- **Infarct Status – Salvagable Areas, Infarct Size, and Infarct Volume-** The stroke lesion volume will be assessed by T2 weighted and FLAIR (fluid-attenuated inversion recovery) MRI or CT scan.

  Measurements will be performed at baseline and will be compared to the a follow up scan performed at at 60 days.
- **Clinical Outcomes - Improvement of Neurological Status**- Neurological assessment using the NIHSS and Modified Rankin scores will be performed.

  NIHSS will be performed at baseline and during the first week; Modified Rankin scale will be performed prior to discharge.

  A weekly assessment including both scales will be performed following discharge during the first 30 days. Additional follow up assessments will be performed at 60 and 90 days post enrollment to the trial.

  During the follow up assessment visits all patients will be subject to additional outcomes assessment scales including the Rankin score and Barthel Assessment of Motor Recovery.

4.5 *Procedural Protocol*

4.5.1 Screening and enrollment

Patients with known acute ischemic stroke within the last 24 hours, who fulfill the eligibility criteria specified for this study (see inclusion/exclusion criteria).

The screening process will include the following:

- Medical history
- Clinical and Neurological history
- NIHSS assessment scoring
- Blood Tests
- Head CT or MRI scan
- Informed Consent explanation, and signature

4.5.2 Baseline measurements

- Physical and neurological examination

  A standard neurological baseline examination shall take place. The neurological examination will include the following:

  - Cognitive function
  - Cranial nerves
  - Strength
  - Deep tendon reflexes
  - Plantar response
  - Sensory
  - Coordination/Gait

- Baseline diffusion-perfusion and T2 weighted and Fluid Attenuated Inversion Recovery (FLAIR) MRI Imaging or CT perfusion – Intended to document a baseline measure of mismatch (DWI–PWI mismatch); perfusion deficit and core volume respectively
- TCD – a recording before surgery should be done to all patients
- Laboratory tests - including ECG, Blood Pressure[5], and blood tests performed at baseline
  - Blood Tests will include:
    - CBC/WBC/differential/platelet count
    - <u>Serum chemistry</u>
      - Blood glucose
      - Serum electrolytes
      - ProThrombin Time (PT) or INR, activated Partial Thromboplastin Time (aPTT)

[5] ECG recordings and BP measurements performed before, during and after treatment sessions will be part of the safety analysis.

4.5.3 Treatment Period

4.5.3.1 NeuroPath™ IS System implantation

Patients classified to the implanted arm should be undergoing the implantation. The NeuroPath**™** IS System will be implanted using a minimal invasive approach. Total procedure time will be ~20 minutes. The stimulation will be performed for a total 7 days, 3 hours daily.

The stimulation parameters of the NeuroPath™ IS System are based upon two primary sources [3,4] (for additional information refer to section 2.7.3). Treatment Period will be performed ONLY during hospitalization.

Measurements and evaluation during hospitalization phase

- Neurological and clinical assessments using standard scales
  - The **NIHSS** will be performed at 24 hours; 3 days post event and on day 7 (i.e. last treatment day).
  - The Modified **Rankin Score** will be performed prior to discharge as the baseline measurement
  - **Barthel** Index Assessment scale will be performed prior to discharge as the baseline measurement
- SPECT scan or CT/MRI perfusion will be performed at day one of treatment phase and on the last day of treatment -7th day.
- TCD Recordings - Transcranial Doppler (TCD) sonography will be performed within 3 treatment sessions; the measurement should be performed so recordings will be gathered prior to stimulation; during stimulation and immediately after stimulation has stopped.
- General clinical examination including ECG, BP and blood tests will be performed in accordance with best medical care practice during hospitalization, and prior to discharge
  - HR and BP will be recorded before, during and one hour post stimulation session. Such measurements will be done at day 1 and at least twice during the hospitalization period.

If not done during the hospitalization period, Patients will be invited for implant removal session; this must be done on between the 7th to 28th days of treatment.

Following that the patients will be invited to the clinic for 'a check up' visit at 48 to 72 hours following implant removal.

4.5.4 Follow Up Period

The period will include at least 4 visits – at 14+/- 2; at 30+/-2; at 60+/-5 and at 90+/-5 days post enrollment; In each of the visit the investigator is required to performed the following assessments:

- General clinical assessment.

- Neurological scales assessments: NIHSS

  Rehabilitation scales assessments: Rankin and Barthel scale
- MRI or CT Scans (T2W and FLAIR) will be performed to all patients on day 60 ± 5

4.5.4.1 Unscheduled Visits

Unscheduled visits should be performed in any case of clinical worsening. In case of visit performed, the following assessments are required:

- A CT or MRI imaging for documented brain status
- A clinical neurological assessment using the NIHSS and mRS / Barthel

5 Adverse Events

5.1 *Definitions*

An adverse event is any undesirable experience (sign, symptom, illness, abnormal laboratory value, or other medical event) occurring to a patient that appears or worsens during a clinical trial. An adverse event may or may not be related to the investigational device prescribed as part of the trial protocol.

All adverse events will be graded as follows:

Mild: Sign or symptom, usually transient, non life-threatening requiring no special treatment and generally not interfering with usual activities.

Moderate: Sign or symptom, non life-threatening which may be ameliorated by simple therapeutic measures, and may interfere with usual activity.

Major: Sign or symptom that is intense or debilitating but non life-threatening and that interferes with usual activities. Recovery is usually aided by therapeutic measures and the discontinuation of the study device may be required.

Severe: Any untoward medical occurrence that at any time results in death or life-threatening illness, resulting in persistent or significant disability/incapacity.

The relationship of the adverse event to the study is defined as follows:

Probable: An adverse event has a strong temporal relationship to study device, and another etiology is unlikely or significantly less likely.

Possible: An adverse event has a strong temporal relationship to the study device, and an alternative etiology is equally or less likely compared to the potential relationship to study device.

Probably not: An adverse event has little or no temporal relationship to the study device and/or a more likely alternative etiology exists.

Not related: An adverse event has no temporal relationship to study device or has a much more likely alternative etiology.

5.2 *Adverse Events Reporting*

Each adverse event will be reported to BrainsGate. The severity scoring should comply with the Common Terminology Criteria for Adverse Events version 3.0 as possible.

Unanticipated adverse events and device related adverse events will be reported immediately (with in **24 hours**) to BrainsGate both by fax on adverse event CRF page and by a telephone call.

Anticipated adverse events will be reported **within five (5) days** both by fax and by a telephone call.

All unanticipated and device related events will once reported to BrainsGate, undergo immediate review BrainsGate. If there is a technical problem that is deemed by BrainsGate, as such that there is a risk for the patient arising from it – all implantations that had not been carried out, will be halted, and all stimulation (if relevant) in patients that had been implanted will be halted, until a satisfactory resolution of the problem which had been reviewed by BrainsGate will be found and approved by BrainsGate. Device related adverse events will be reported to the all participant centers' Ethical committees promptly.

If the conclusion of BrainsGate is that there should be a change in the protocol as a result of the event reported as needed, it will be changed as soon as possible according to the regulations. If BrainsGate deems the problem to require immediate changes, all treatments will be suspended until such a change will take place. Events will be reported to the Ethical committee and to the local health authorities according to the rules and regulations.

In case of a serious or severe adverse event or in case of other emergency need the following contact should be used:

Adar Shani – Clinical Manager, BrainsGate Ltd.

Tel: +972-9-7456252 ext. 112

Cell: +972-54-2347770

Subhash Sethi – Wellness Concepts Consulting

Tel: +91-9820284121

Cell: +91-22-56872142

6 Statistical Analysis

6.1 *Rationale*

This Pilot/safety and feasibility pilot study will comprise of 30 patients.

The study will be considered successful if a low incidence of procedure complications, expected/ unexpected adverse events and SAE.

Secondary end points will be considered successful if a substantial percentage of the patients will show augmentation of cerebral brain perfusion during the whole length of the treatment protocol and improvement of neurological status can be demonstrated in those patients population.

6.2 *Interim Analysis*

As this study is non-statistical in nature, no statistical interim analysis will be performed and therefore there will be no early termination on statistical grounds.

6.3 *Patient Incorporation Policy*

All eligible participants in this study will be included in the analysis, which will not be statistical in nature due to the characteristic of the study.

6.4 *Data Accountability*

All the relevant clinical data and imaging will be collected from the study site by BrainsGate representative or BrainsGate designee during routine monitoring visits. These visits will be carried out to coincide with the implantation and the stimulation, and thereafter the follow-up data will be collected in less frequent monitoring visits. During those visits, all efforts will be made to complete missing data according to regulations. However, missing data, will not, if at all possible, exclude the patient from analysis.

7 INVESTIGATION ADMINISTRATION

7.1 *Study Monitoring*

Monitoring functions shall be performed in compliance with 21CFR§812.43(d) and 21CFR§812.46

The major function of the clinical monitor is to observe and assess the quality of the clinical study. For this study, qualified personnel will be appointed by BrainsGate Ltd.

Apart from assuming responsibility for the communication between the investigators and BrainsGate (BrainsGate Ltd..), the monitor's duties include on-site visits, participation in initial study sessions, review of Case Report Forms and other study documents and results.

7.2 *On-Site Visits*

On-site monitoring visits include pre-study visit, periodic visits, and final visit at the close of the study.

The pre-study visit is intended to review with the investigator the final approved version of the protocol and to assure that the investigator has obtained institutional approval for conducting the study, and has all study documentation, required records, and equipment on site.

Periodic visits are intended to assess adherence to the protocol, maintenance of records, reports and devices, and review of source documents for accuracy, completeness, and legibility.

During these periodic visits the monitor is required to assess the progress of the study toward meeting study objectives and identify any concerns that stem from observations and review of patient records, study management documents, informed consent documents, and adverse events records.

At the close of the study, the monitor will make a final on-site visit to ensure that all study data has been properly completed and to have a closing meeting with the investigator and medical staff.

Reports of on-site visits should be generated by the monitor, as needed to inform BrainsGate of required corrective actions, resolution of concerns, completion of follow-up activities, and completion of assigned tasks.

At the close of the study, the monitor is required to prepare and provide with BrainsGate a final monitoring report.

7.3 *Case Report Forms*

Case Report Forms will be provided for each patient. On completion, Case Report Forms must be reviewed for completeness and accuracy, dated, and signed by the investigator.

Crossing out the incorrect information shall only make corrections of data on the Case Report Forms and writing the correct values next to those crossed out. Each correction must be initiated and dated by the investigator or an authorized assistant. In no instance may data be altered by use of correction fluid, erasures, etc.

*Case report forms will be sent via facsimile to BrainsGate or to its local representative every monitoring visit with an attached cover page.

7.4 *Review of Study Documents*

The monitor will review completed data forms and study documentation for accuracy, completeness, and protocol compliance. In particular, the monitor should:

(1) Compare the Case Report Forms to original (source) documents to adequately evaluate accuracy and completeness of data.

(2) Review all Case Report Forms for errors, omissions, internal consistency, and signature and dates in the appropriate sections.

(3) Verify that a patient’s written Informed Consent has been duly completed and signed for each subject prior to his/her participation in the study.

(4) Assume responsibility for any follow-up activities that result from review of study forms and documents.

7.5 *Patient Confidentiality*

All reports and communications relating to study subjects will identify the subject only by his/her trial I.D code. The investigator will complete subject identification on a confidential patient log, which will be used for purposes of patient tracking and follow-up. This will be treated in accordance with strict adherence to professional standards of confidentiality, and will be filed with adequate security and restricted accessibility.

Signed Informed Consent Forms bearing the full name and signature of patients represent confidential documents and should therefore be archived in the Investigator File.

7.6 *Study Discontinuation*

The study may be discontinued if:

a) At any time, in the opinion of the Institutional Ethical Committee, the investigator, and/or the company, the study represents an unreasonable medical risk to patients.

b) The Sponsor decides to terminate the study due to company considerations.

7.7 *Protocol Amendments*

The protocol is not to be modified by the investigator without first obtaining review and agreement of BrainsGate.

Medically significant amendments to the protocol (e.g., changes that increase the risk or the inconveniences for the patient, inclusion of new categories of patients, etc.) must be approved by the local Ethical Committee prior to implementation.

7.8 *Study Documentation and Data Management*

The following data will be recorded pre, during and post-procedure on the Case Report Forms:

(1) BASELINE FORM - to be used for screening patients for inclusion into the study and to record patient's demographic data as well as patient's medical history.

(2) PROCEDURE FORM - to be used to record procedural details, procedural complications, technical aspects of the system, and other relevant information related to the procedure.

(3) FOLLOW-UP FORM – to be used during the standard post-procedure checkup visit at the clinic to record the current condition of the patient, and occurrence of post-procedural complication if any.

(4) ADVERSE EVENT FORM – to be used for recording any complication that occurred during the study.

(5) EXIT FORM – to be used to record completion of study, pre-mature withdrawals, and/or lost to follow up cases.

All data collected in the CRF will be stored in a computer form (database). Data will be entered into a commercially available or validated system, using a double data entry (DDE) method. In case of changes to the data, the system should maintain an audit trial of all the changes.

The investigator will be responsible for keeping the following records:

- Ethical committee Approval
- Signed "Certification: Financial Interests and Arrangements of Clinical Investigators"
- Signed Informed Consent Forms
- Correspondence related to the study
- Completed Patient Log
- Completed Case Report Forms
- Any additional records and reports required by the Hospital and/or Regulatory Authorities

7.9 *Records Retention*

The investigator will arrange for the retention of these documents for a period of no less than three years after the latter of the following dates: the date of which the study is terminated or completed or; the date that the records are no longer required supporting marketing applications. The investigator should not relocate or dispose of any study documents before having obtained BrainsGate written permission.

8 APPENDICES

8.1 *Appendix A – Study Flow Chart*

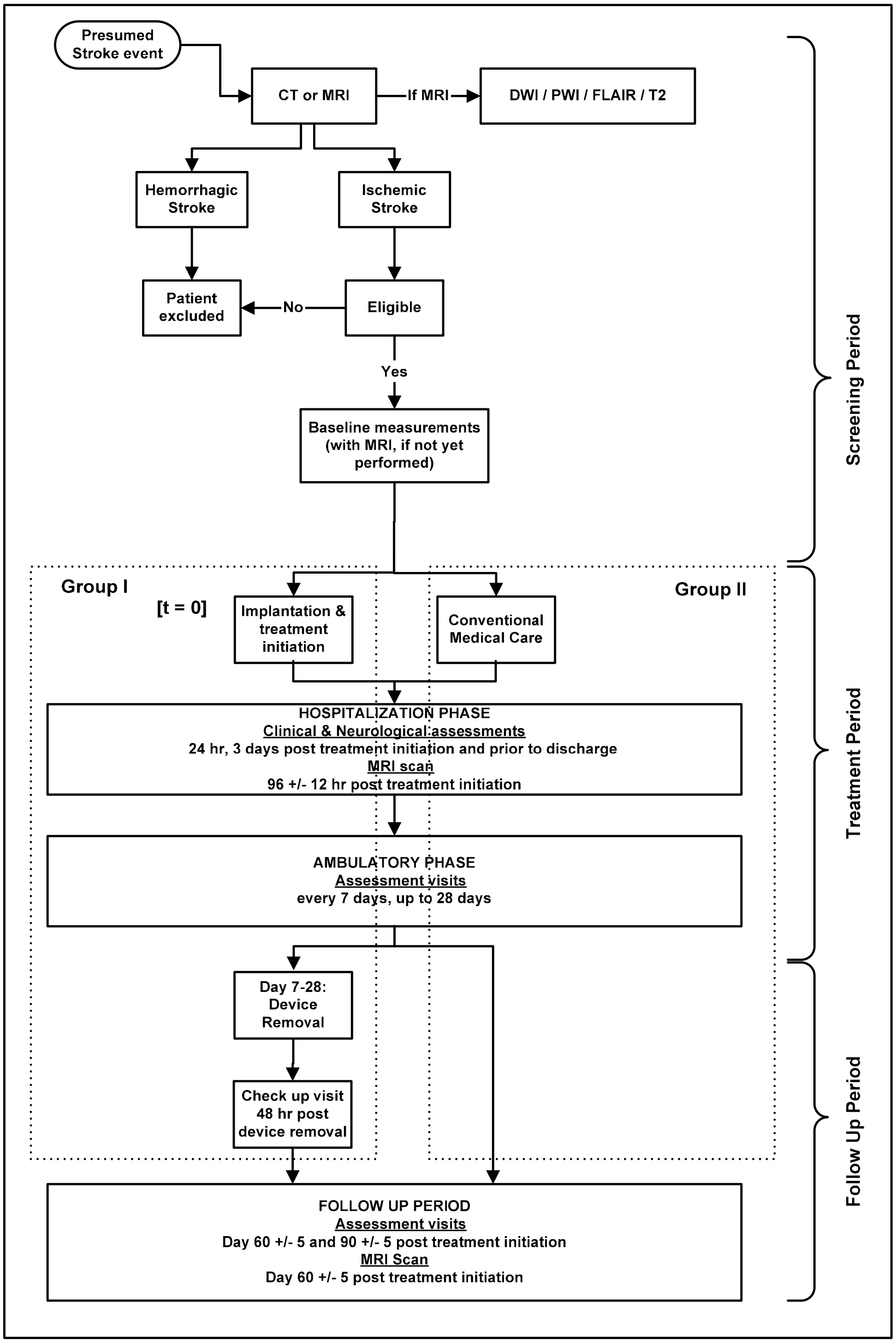

8.2 *Appendix B - Trial measurements schematic*

	Eligibility	Baseline		24 hours post E	3 days post E	96 hours (+12 hrs) post E	7 days post E (end of treatment)	On discharge	Day 30 ± 5 post E	Day 60 ± 5 post E	Day 90 ± 5 post E
**MRI Imaging** **DWI /PWI , T2 weighted and Fluid Attenuated Inversion Recovery (FLAIR)**	+		**Enrollment**			+				+	
**NIHSS**		+		+	+		+	+	+	+	+
**Modified Rankin Score**							+	+	+	+	+
**Barthel**							+	+	+	+	+
**SPECT scan**				+ Before and after the stimulation procedure							
**TCD measurement**				At least 3 times during the treatment session							
**HR/ BP**		+		+	+ At least one additional time						
**Blood Test**		+			+ At least one additional time						
**Implant removal** (Between 8-28 days)							+				

REFFERANCES

1. Murray CJ, Lopez AD. Mortality by cause for eight regions of the world: Global Burden of Disease Study. Lancet 1997; 349: 1269-76.
2. American Heart Association: Heart disease and stroke statistics - 2005 update. Dallas, Texas: American Heart Association, 2005.
3. Broderick J, Brott T, Kothari R, et al.: The Greater Cincinnati/Northern Kentucky Stroke Study: preliminary first-ever and total incidence rates of stroke among blacks. Stroke, vol 29, 1998; 415-21.
4. Williams GR. Incidence and characteristics of total stroke in the United States. BMC Neurol 2001; 1: 2.
5. Rothwell PM, Coull AJ, Silver LE, et al. Population-based study of event-rate, incidence, case fatality, and mortality for all acute vascular events in all arterial territories (Oxford Vascular Study). Lancet 2005; 366: 1773-83.
6. Steiner A, Neu C: Monitoring the change in use of Medicare post-hospital services.: UCLA-Harvard Center for Health Care Financing Policy Research, 1993.
7. Black-Schaffer RM, Osberg JS. Return to work after stroke: Development of a predictive model. Arch Phys Med Rehab 1990; 71: 285-290.
8. Anderson CS, Linto J, Stewart-Wynne EG. A population-based assessment of the impact and burden of caregiving for long-term stroke survivors. Stroke 1995; 26: 843-849.
9. Day J: Population projections of the United States by age, sex, race, and Hispanic origin: 1995 to 2050. U.S. Government Printing Office, Washington, DC: U.S. Bureau of the Census, Current Population Reports, 1996.
10. Feigin VL, Lawes CM, Bennett DA, Anderson CS. Stroke epidemiology: a review of population-based studies of incidence, prevalence, and case-fatality in the late 20th century. Lancet Neurol 2003; 2: 43-53.
11. Weinstein PR, Hong S, Sharp FR. Molecular identification of the ischemic penumbra. Stroke 2004; 35: 2666-2670.
12. Latchaw RE, Yonas H, Hunter GJ, et al. Guidelines and recommendations for perfusion imaging in cerebral ischemia: A scientific statement for healthcare professionals by the writing group on perfusion imaging, from the Council on Cardiovascular Radiology of the American Heart Association. Stroke 2003; 34: 1084-104.
13. Wintermark M, Sesay M, Barbier E, et al. Comparative overview of brain perfusion imaging techniques. Stroke 2005; 36: e83-99.
14. Markus R, Reutens DC, Kazui S, et al. Hypoxic tissue in ischemic stroke: persistence and clinical consequences of spontaneous survival. Brain 2004; 127: 1427-1436.
15. Darby DG, Barber PA, Gerraty RP, et al. Pathophysiological topography of acute ischemia by combined diffusion-weighted and perfusion MRI. Stroke 1999; 30: 2043-2052.
16. The National Institute of Neurological Disorders and Stroke rt-PA Stroke Study Group. Tissue Plasminogen Activator for acute ischemic stroke. N Engl J Med 1995; 333: 1581-1588.
17. Kwiatkowski TG, Libman RB, Frankel M, et al. Effects of tissue plasminogen activator for acute ischemic stroke at one year. National Institute of Neurological Disorders and Stroke Recombinant Tissue Plasminogen Activator Stroke Study Group. N Engl J Med 1999; 340: 1781-7.

18. Tanne D, Kasner SE, Demchuk AM, et al. Markers of increased risk of intracerebral hemorrhage after intravenous recombinant tissue plasminogen activator therapy for acute ischemic stroke in clinical practice: the Multicenter rt-PA Stroke Survey. Circulation 2002; 105: 1679-85.
19. Graham GD. Tissue plasminogen activator for acute ischemic stroke in clinical practice: A meta-analysis of safety data. Stroke 2003; 34: 2847-2850.
20. Hill MD, Buchan AM. Thrombolysis for acute ischemic stroke: results of the Canadian Alteplase for Stroke Effectiveness Study. Cmaj 2005; 172: 1307-12.
21. Lisboa RC, Jovanovic BD, Alberts MJ. Analysis of the safety and efficacy of intra-arterial thrombolytic therapy in ischemic stroke. Stroke 2002; 33: 2866-2871.
22. Furlan A, Higashida R, Wechsler L, et al. Intra-arterial prourokinase for acute ischemic stroke. The PROACT II study: a randomized controlled trial. Prolyse in Acute Cerebral Thromboembolism. Jama 1999; 282: 2003-11.
23. Smith WS, Sung G, Starkman S, et al. Safety and efficacy of mechanical embolectomy in acute ischemic stroke: results of the MERCI trial. Stroke 2005; 36: 1432-1438.
24. Liebeskind DS. Collateral circulation. Stroke 2003; 34: 2279-2284.
25. Coyle P, Heistad DD. Development of collaterals in the cerebral circulation. Blood Vessels 1991; 28: 183-189.
26. Wang CX, Todd KG, Yang Y, Gordon T, Shuaib A. Patency of cerebral microvessels after focal embolic stroke in the rat. Journal of Cerebral Blood Flow and Metabolism 2001; 21: 413-421.
27. Wei L, Erinjeri JP, Rovainen CM, Woolsey TA. Collateral growth and angiogenesis around cortical stroke. Stroke 2001; 32: 2179-2184.
28. Rordorf G, Koroshetz WJ, Ezzeddine MA, Segal AZ, Buonanno FS. A pilot study of drug-induced hypertension for treatment of acute stroke. Neurology 2001; 56: 1210-1213.
29. Hillis AE, Ulatowski JA, Wityk RJ, et al. A pilot randomized trial of induced blood pressure elevation: Effects on function and focal perfusion in acute and subacute stroke. Cerebrovascular Diseases 2003; 16: 236-246.
30. Schwarz S, Georgiadis D, Aschoff A, Schwab S. Effects of induced hypertension on intracranial pressure and flow velocities of the middle cerebral arteries in patients with large hemispheric stroke. Stroke 2002; 33: 998-1004.
31. Edvinsson L, Hamel E. Perivascular nerves in brain vessels. Cerebral Blood Flow and Metabolism 2002: 43-67.
32. Toda N, Tanaka T, Ayajiki K, Okamura T. Cerebral vasodilatation induced by stimulation of the pterygopalatine ganglion and greater petrosal nerve in anesthetized monkeys. Neuroscience 2000; 96: 393-398.
33. Hara H, Zhang Q-J, Kuroyanagi T, Kobayashi S, Selman W, Findlay JM. Parasympathetic cerebrovascular innervation: An anterograde tracing from the sphenopalatine ganglion in the rat. Neurosurgery 1993; 32: 822-827.
34. Suzuki N, Hardebo JE, Kahrstrom J, Owman C. Selective electrical stimulation of postganglionic cerebrovascular parasympathetic nerve fibers originating from the sphenopalatine ganglion enhances cortical blood flow in the rat. J. Cereb. Blood Flow Metab. 1990; 10: 383-391.
35. Suzuki N, Hardebo JE, Skagerberg G, Owman C. Central origins of preganglionic fibers to the sphenopalatine ganglion in the rat. A fluorescent retrograde tracer study with special reference to its relation to central catecholaminergic systems. Journal of the Autonomic Nervous System 1990; 30: 101-109.

36. Toda N, Ayajiki K, Tanaka T, Okamura T. Preganglionic and postganglionic neurons responsible for cerebral vasodilation mediated by nitric oxide in anesthetized dogs. Journal of Cerebral Blood Flow and Metabolism 2000; 20: 700-708.
37. Seylaz J, Hara H, Pinard E, Mraovitch S, MacKenzie ET, Edvinsson L. Effect of stimulation of the sphenopalatine ganglion on cortical blood flow in the rat. Journal of Cerebral Blood Flow and Metabolism 1988; 8: 875-878.
38. Goadsby PJ, Uddman R, Edvinsson L. Cerebral vasodilatation in the cat involves nitric oxide from parasympathetic nerves. Brain Research 1996; 707: 110-118.
39. Edvinsson L, Fahrenkrug J, Hanko J, et a. VIP (vasoactive intestinal polypeptide)-containing nerves of intracranial arteries in mammals. Cell Tissue Res. 1980; 208: 135-142.
40. Goadsby PJ, Edvinsson L: Neurovascular control of the cerebral circulation. In: Edvinsson L, Krause D, eds. Cerebral blood flow and metabolism. Philadelphia: Lippincott, Williams & Wilkins, 2002; 172-188.
41. Toda N, Ayajiki K, Yoshida K, Kimura H, Okamura T. Impairment by damage of the pterygopalatine ganglion of nitroxidergic vasodilator nerve function in canine cerebral and retinal arteries. Circ. Res. 1993; 72: 206-213.
42. Ayajiki K, Fujioka H, Shinozaki K, Okamura T. Effects of capsaicin and nitric oxide synthase inhibitor on increase in cerebral blood flow induced by sensory and parasympathetic nerve stimulation in the rat. Journal of Applied Physiology 2005; 98: 1792-1798.
43. Lee TJF. Nitric oxide and the cerebral vascular function. Journal of Biomedical Science 2000; 7: 16-26.
44. Brayden J, Bevan J. Evidence that vasoactive intestinal polypeptide (VIP) mediates neurogenic vasodilation of feline cerebral arteries. Stroke 1986; 17: 1189-1192.
45. Okamura T, Ayajiki K, Fujioka H, Shinozaki K, Toda N. Neurogenic cerebral vasodilation mediated by nitric oxide. Japanese Journal of Pharmacology 2002; 88: 32-38.
46. Toda N, Okamura T. The pharmacology of nitric oxide in the peripheral nervous system of blood vessels. Pharmacological Reviews 2003; 55: 271-324.
47. Willmot M, Gray L, Gibson C, Murphy S, Bath PMW. A systematic review of nitric oxide donors and L-arginine in experimental stroke; effects on infarct size and cerebral blood flow. Nitric Oxide - Biology and Chemistry 2005; 12: 141-149.
48. Cooke JP. NO and angiogenesis. Atherosclerosis Supplements 2003; 4: 53-60.
49. Estrada C, Murillo-Carretero M. Nitric oxide and adult neurogenesis in health and disease. Neuroscientist 2005; 11: 294-307.
50. Zhang R, Zhang L, Zhang Z, et al. A nitric oxide donor induces neurogenesis and reduces functional deficits after stroke in rats. Annals of Neurology 2001; 50: 602-611.
51. Chen J, Li Y, Zhang R, et al. Combination therapy of stroke in rats with a nitric oxide donor and human bone marrow stromal cells enhances angiogenesis and neurogenesis. Brain Research 2004; 1005: 21-28.
52. Yarnitsky D, Lorian A, Shalev A, et al. Reversal of cerebral vasospasm by sphenopalatine ganglion stimulation in a dog model of subarachnoid hemorrhage. Surgical Neurology 2005; 64: 5-11.
53. Glenn D. Graham, MD, PhD; Tissue Plasminogen Activator for Acute Ischemic Stroke in Clinical Practice. Stroke. 2003;34:2847

54. Zhang R, Zhang L, Zhang Z, Wang Y, Lu M, Lapointe M, Chopp M. A nitric oxide donor induces neurogenesis and reduces functional deficits after stroke in rats. Ann Neurol. 2001 Nov;50(5):602-11
55. Badtke and Mudra; Neuraltherapie; Lehrbuch und Atlas Ullstein Medical 1998, pg 108-109.
56. Suzuki N, Hardebo JE; Anatomical basis for parasympathetic and sensory innervation of the intracranial segment of the internal carotid artery in man., J Neurolo Sci 1991 July 104 (11) 19-31
